# Disruption of C/EBPβ-Clec7a axis exacerbates neuroinflammatory injury via NLRP3 inflammasome-mediated pyroptosis in experimental neuropathic pain

**DOI:** 10.1186/s12967-022-03779-9

**Published:** 2022-12-12

**Authors:** Dan Wu, Yanqiong Zhang, Chunhui Zhao, Qiuyue Li, Junhong Zhang, Jiaxin Han, Zhijian Xu, Junfang Li, Yan Ma, Ping Wang, Haiyu Xu

**Affiliations:** 1 Institute of Chinese Materia Medica, China Academy of Chinese Medical Sciences, Beijing, 100700 China; 2 Drug Discovery and Design Center, Shanghai Institute of Materia Medica, Chinese Academy of Sciences, Shanghai, 201203 China; 3 Key Laboratory for Research and Evaluation of Traditional Chinese Medicine, National Medical Products Administration, China Academy of Chinese Medical Sciences, Beijing, 100700 China

**Keywords:** C/EBPβ-Clec7a axis, Neuroinflammation, Neuropathic Pain, NLRP3 Inflammasome-mediated Pyroptosis

## Abstract

**Background:**

Growing evidence shows that C-Type Lectin Domain Containing 7A (Clec7a) may be involved into neuroinflammatory injury of various neurological diseases. However, its roles in neuropathic pain remain unclear.

**Methods:**

A chronic constriction injury (CCI) rat model was constructed, and gene expression profilings in spinal cord tissues of CCI-insulted rats were detected by both microarray and RNA-seq studies. A series of bioinformatics analyses identified C/EBPβ-Clec7a to be a candidate axis involved into neuropathic pain. Then, its roles in mechanical allodynia, and pathological and molecular changes during CCI progression were determined by various gain-of-function and loss-of-function experiments in vivo and in vitro.

**Results:**

Significant upregulation of Clec7a at both mRNA and protein levels were verified in spinal cord tissues of CCI-insulted rats. Clec7a knockdown markedly attenuated CCI-induced mechanical allodynia, obstructed Syk, ERK and JNK phosphorylation, inhibited NLRP3 inflammasome and caspase-1 activation, GSDMD cleavage, and consequently reduced the release of pro-inflammatory cytokines (all *P* < 0.05). Mechanically, the rat Clec7a promoter was predicted to bind with transcription factor C/EBPβ, confirmed by Luciferase assay and ChIP-qPCR. Both in vivo and in vitro assays demonstrated that C/EBPβ knockdown significantly suppressed CCI- or LPS/ATP-induced Clec7a upregulation, and subsequently reduced Syk, ERK and JNK phosphorylation, NLRP3 oligomerization, caspase-1 activation, GSDMD expression and pyroptosis, which were markedly reversed by the co-transfection of Clec7a expression vector.

**Conclusions:**

This pre-clinical investigation reveals that C/EBPβ-Clec7a axis may be a potential target for relieving neuropathic pain through alleviating neuroinflammation, paving its way for clinical translation as a promising approach for neuropathic pain therapy.

**Supplementary Information:**

The online version contains supplementary material available at 10.1186/s12967-022-03779-9.

## Background

Neuropathic pain is a complex chronic pain due to the nervous system damage or diseases, including cancer, stroke, infection, diabetic neuropathy, spinal cord injury, or degenerative neurological diseases [1, 2]. During neuropathic pain development and progression, the neuroinflammation has been observed along the pain pathways from the spinal cord to the thalamus and the parietal cortex [3]. It may be caused by the activation of glial cells, especially microglia, with production of cytokines and other inflammatory mediators within the central nervous system [4]. Growing evidence shows that neuroinflammation may enhance pain states and promote pain spreading [5]. Therefore, it is of significance to identify novel targets for neuropathic pain therapy by attenuating neuroinflammation and restoring glial cell function.

Beyond the canonical types of cellular apoptosis and necrosis, pyroptosis has been revealed as a novel type of regulated cell death [6]. During the occurrence of pyroptosis, pattern recognition receptors may induce the activation of cysteine-aspartic protease 1 (caspase-1) or caspase-11, which can trigger the release of the pyrogenic cytokines interleukin-1β (IL-1β) and IL-18 [7]. Recent studies have observed that some damaged neurocytes associated with neuropathic pain may exhibit pathological changes similar to pyroptosis, implying that this type of regulated cell death may contribute significantly to the pathology of neuropathic pain [8]. Of note, a previous study of Wang et al. [9] reported that the spinal nerve ligation modeling significantly up-regulated the inflammatory cytokines associated with the excessive activation of NOD-like receptor family pyrin domain-3 (NLRP3) components and NF-κB signaling, as well as a marked pyroptosis activation. However, the involvement of pyroptosis in neuropathic pain development and progression, and its therapeutic potentials have not fully elucidated.

Both hybridization-based microarray and RNA sequencing (RNA-seq) studies are commonly used to understand gene expression patterns in various physiological and pathological conditions. Accumulating studies have revealed that the dramatic transcriptional changes of many genes in spinal cord (SC) may drive neuropathic pain development and progression after nerve injury [10–12], implying that high-throughput transcriptome analyses have been successfully applied in identifying neuropathic pain-associated gene-expression signatures, which may be candidate markers for the diagnosis and treatment of neuropathic pain. In the current study, chronic constriction injury (CCI) rat model was established by loosely tying the sciatic nerve with 4 ligatures (4–0 chromic catgut) at the mid-thigh level. Gene expression profiling in SC tissues of the CCI and Sham groups were obtained from both microarray and RNA-seq studies. Following differentially expressed gene screening, bioinformatics prediction of the interaction of transcription factor-target gene and coexpression analysis, a candidate transcription factor-target gene axis involved into NLRP3 Inflammasome-mediated pyroptosis during neuropathic pain progression was selected. Then, its roles in mechanical allodynia of CCI rats, as well as pathological and molecular changes in pyroptosis during CCI progression were determined by various gain-of-function and loss-of-function experiments in vivo and in vitro, including luciferase assays, ChIP-qPCR, RNA interference (siRNA), western blotting, immunofluorescence, immunohistochemistry, tunel staining and enzyme-linked immunosorbent assay (ELISA).

## Methods

### Ethics statement

All experimental protocols and animal-handling procedures were approved by our institutional Animal Care Committee (No. IACUC-AEWC-F2010007, F2101010, F2105007) and were performed in accordance with the recommendations outlined in the Guide for the Care and Use of Laboratory Animals published by the National Institutes of Health.

### Animals and grouping

A total of 210 adult male Sprague–Dawley rats (6–7 weeks, weigh 180–220 g) were purchased from Beijing Vital River Laboratory Animal Technology Co. Ltd. Rats were housed with a fixed light/dark cycle (12/12 h), a room temperature of 21− 22 ℃, and ad libitum access to food and water. After adaptive feeding for 1 week, rats were randomly divided into different groups. All experiments were designed to minimize the number of animals used and their suffering.

In microarray and RNA-seq, a total of 14 rats were randomly divided into Sham and CCI groups, 7 rats per group. Among them, 3 rats were used for microarray analyses, and 4 rats for RNA sequencing. In addition, there were 12 rats randomly assigned to the two groups to verify the results of microarray and RNA-seq, 6 rats per group.

Moreover, a total of 100 rats were applied for experiments to explore and validate the potential mechanisms, including knockdown of Clec7a and C/EBPβ. There were 50 rats in each experiment randomly assigned to five groups: (1) Sham group; (2) Sham + si-*NC* group; (3) CCI group; (4) CCI + si-*NC* group; (5) CCI + si-*Clec7a* or si-*Cebpb* group, with 10 rats in each group.

In add-back rescue experiments, a total of 84 rats were randomly divided into six groups, including 9 rats in Sham group and 15 rats in other five groups (CCI group, CCI + pLV.1-NC + pLVSO5-NC group, CCI + pLV.1-sh*Cebpb* + pLVSO5-NC group, CCI + pLV.1-NC + pLVSO5-*Clec7a-*overexpress group and CCI + pLV.1-sh*Cebpb* + pLVSO5- *Clec7a-*overexpress group).

### CCI model

The CCI rat model was established as previously described [13]. Rats were anaesthetised with isoflurane. Briefly, the left common sciatic nerve in each rat was exposed by blunt dissection. The area proximal to the sciatic nerve was free of adhering tissue, and four ligatures were tied loosely around it with approximately 1 mm interval. The sciatic nerve was exposed but not ligated in Sham group animals.

### Mechanical allodynia analyses

Mechanical allodynia was measured using the von Frey test (ALMEMO 2390, IITC Life Science, USA) at 1 day before the surgical procedure at days 1, 3, 5, 7 and 9, respectively, after sciatic nerve injury. Rats were placed individually in a transparent plexiglass box on a 50 cm high shelf, and the top of the shelf is a wire mesh with small holes. Rats were acclimated to the test environment at least 20 min. After the rats adapt to being quiet, Von-Frey was applied to the plantar middle of the rat hind paw through the wire mesh for 4–6 s. It is measured once every 30 s for 5 times. The data of mechanical withdrawal threshold (MWT) were shown as the mean of five readings.

### Gene expression profiling & data analysis

L_4_-L_6_ SC tissues collected from the rats in the Sham and CCI groups on postoperative day 10 were immersed in TRIzol (Invitrogen, CA, USA) and immediately frozen in liquid nitrogen for both the whole-genome microarray (n = 3 per group and mixed one sample for the detection) and RNA-seq (n = 4 per group) studies. Microarray detection was performed using the Affymetrix GeneChip^®^ Rat Gene 2.0 ST Arrays (Affymetrix, Santa Clara, CA) by Shanghai GMINIX Biotechnology Corporation (Shanghai, China). RNA-sequencing was carried out by the Novogene Bioinformatics Technology Co.Ltd (Beijing, China). Sequencing libraries were generated using NEBNextUltra RNA Library Prep Set for Illumina (NEB, USA.) following manufacturer’s recommendations. The clustering of the index-coded samples was performed on a cBot Cluster Generation System using TruSeq SR Cluster Kit v3-cBot-HS (Illumia) according to the manufacturer’s instructions. After cluster generation, the library preparations were sequenced on an Illumina Hiseq 2500/2000 platform. Gene expression profiling was analyzed using R (http://www.rproject.org/, version 3.1.1) with Bioconductor packages (http://www.bioconductor.org/). Raw intensities were normalized using Robust multi-array average (RMA method) [14]. All raw data were be deposited in GEO datasets (https://www.ncbi.nlm.nih.gov/geo/query/acc.cgi?acc=GSE186237 and https://www.ncbi.nlm.nih.gov/geo/query/acc.cgi?acc=GSE185278). Significant differentially expressed genes (DEGs) of the Sham vs. CCI groups were identified using the criteria of a |fold change|> 1.5 and *P* value < 0.05.

### Small interfering RNA (siRNA) and transfection

Clec7a siRNA (si-*Clec7a*), C/EBPβ siRNA (si-*Cebpb*) and control siRNAs (si-NC) designed and synthesized by RiboBio (Guangzhou, China) were separately dissolved in Opti-MEM (Gibco, USA). After 5 min of equilibration at room temperature, each siRNA solution was combined with the respective volume of the transfection reagent, mixed gently and incubated for 20 min. BV2 cells were transfected with the transfection mixture in Opti-MEM for 6 h.The cells were then changed to antibiotic-free medium and continued culturing for 48 h before follow-up experiments.

To transfect siRNA, Lipofectamine 2000 (Invitrogen, USA) and in *vivo* RNA Advanced Transfection (Zeta Life, USA) were used for BV2 cells and rats, respectively. The sequences of siRNAs are provided in Additional file 2: Table S1.

### Intrathecal catheters

siRNA solution was injected into the unilateral L_4_–L_6_ SC on postoperative day 4 at 2 µg in 10 µL, with the use of a glass micropipette connected to a Hamilton syringe. After each injection, a 5 min pipette retention was used before the glass pipette was removed. The expression levels of Clec7a and C/EBPβ proteins following the specific siRNA transfection were detected by Western blot.

### Cell culture and simulation

Mouse microglial BV2 cell lines were maintained at 37 ℃ in a humidified atmosphere of 5% CO_2_ in DMEM medium (Hyclone, Logan, UT, USA) supplemented with 10% fetal bovine serum (Gibco, Grand Island, NY, USA), 100 IU ml^−1^ penicillin, and 10 μg ml^−1^ streptomycin (Invitrogen, Waltham, MA).

The BV2 cells were adjusted to 5 × 10 [5] cells well^−1^ and were seeded in a 6-well plate for 24 h, and then treated with 0.2 μg mL^−1^ LPS (Sigma, Germany) and 3 mM ATP (Sigma, Germany) for 4 h to establish pyroptosis model.

### Generation of stable cell line inducing C/EBPβ knockdown

The *Cebpb*-shRNA was purchased from General Biosystems (Anhui, China). The pLV.1-sh*Cebpb* recombinant plasmid was constructed by HYY medical science Co.,Ltd (Guangdong, China). Briefly, the shRNA sequences and their antisense sequences were cloned separately into the pLV-CMV-GFP-Puro lentiviral vector, downstream of the CMV promoter. The empty vector was used as the control. BV2 cells were infected with pLV.1-*Cebpb* or control vectors with a multiplicity of infection (MOI) of 50 to 100. Then stable transfectants were screened using 2 μg ml^−1^ puroMycin (Gibco, USA) to generate BV2-pLV.1-shCtrl and BV2-pLV.1-sh*Cebpb* stable cell lines, respectively. The target sequences for sh*Cebpb* was provided in Additional file 2: Table S1.

### Vector construction and lentivirus infection

shRNAs targeting C/EBPβ (sh*Cebpb*) as well as a negative control (shNC) were provided by HYY medical science Co.,Ltd (Guangdong, China). The sh*Cebpb* and shNC were amplified and cloned into LV.1-copGFP to generate pLV.1-shC*ebpb* and pLV.1-shNC. The coding sequences of rat *Clec7a* were amplified and cloned into LVSO5-copGFP (lentivirus gene overexpression vector) to generate pLVSO5-*Clec7a* successfully. The empty pLVSO5 plasmid was used as a negative control. All constructs were verified by DNA sequencing. The packaging lentivirus and target vector were co-transfected into HEK293T cells and cultured for 48 h. The supernatant was then collected, and the lentivirus particles in the supernatant were filtered to detect virus titer. The 5 μL of concentrated lentivirus particles (1X10^9^ transducing/mL) were administrated intrathecally following 1 week of CCI model construction.

### Quantitative real-time PCR

Gene expression levels of *Clec7a* in BV2 cells in different groups were detected using quantitative real-time PCR with a TaqMan Gene Expression Assay by CFX96TM Real-Time Systems according to the manufacturer’s instructions (Bio-Rad, Hercules, CA, USA). Total RNA was extracted using TRNzol Universal reagent (TIANGEN, DP424) according to the manufacturer’s protocol. RNA concentration was determined by a spectrophotometer at 260 nm and 280 nm. Identical amounts of RNA (2 µg) were reverse transcribed into complementary DNA (cDNA) using a FastKing cDNA synthesis kit (TIANGEN, KR118) according to the manufacturer’s instructions. Relative mRNA levels were calculated using the 2^−ΔΔCT^ method [14]. The sequences of primers are provided in Additional file 2: Table S1.

### Western blot analysis

Total proteins were extracted from the L_4_–L_6_ spinal cord segment and BV2 cells in different groups using the RIPA lysis buffer(containing 1 mM phenyl-methylsulfonyl fluoride and protease inhibitor), and the protein concentration was measured using the bicinchoninic acid (BCA) protein concentration test kit (Beyotime, Shanghai, China). Equivalent protein amounts were analyzed on 11% and 8% SDS–PAGE and transferred onto polyvinylidene fluoride (PVDF, Millipore) membrane. The membranes were blocked in non-fat dried milk solution(5% in phosphate-buffer saline) at room temperature for 1.5 h, and then incubated at 4 ℃ overnight with primary antibodies. After washing with 1XTBST, the membrane was incubated with horseradish peroxidase (HRP)-conjugated secondary antibodies at room temperature for 1 h. Bands were visualized with electrochemiluminescence detection reagents. Band intensity was quantified using the Image-Pro Plus 6.0 (Media Cybernetics, Rockville, MD, USA). The detailed information of antibodies used in this study is provided in Additional file 2: Table S2.

### Immunohistochemistry

Following 10 or 30 days of CCI model construction, the L_4_–L_6_ spinal cord segments were obtained, then was fixed overnight in 4% paraformaldehyde, subjected to gradient dehydration, and paraffin-embedded. Sagittal serial sections with a slice thickness of 3 μm were generated. Antigen retrieval was performed by heating the slides at 96 ℃ in sodium citrate buffer (pH 6.0) for 15 min. After cooling to room temperature, slides were rinsed in 0.01 M phosphate-buffered saline (PBS), treated with 0.1% Triton X-100 PBS for 30 min, and washed with 0.01 M PBS three times for 3 min each. Subsequently, endogenous peroxidase was quenched using 3% hydrogen peroxide in PBS for 15 min. Sections were then washed with 0.01 M PBS three times for 8 min each and incubated with specific primary antibodies of Clec7a and C/EBPβ at 4 ℃ overnight. After washing in PBS, sections were incubated for 30 min with a secondary antibody at room temperature. Immunoreactivity was developed with PowerDAB for 7 min at room temperature. Subsequently, sections were counterstained with hematoxylin, rinsed in tap water, dehydrated in 100% ethanol and xylene, and mounted with Permount. The expression levels of Clec7a and C/EBPβ proteins among these sections were evaluated by mean optical density (integrated option density/area) using the Image-Pro Plus 6.0 (Media Cybernetics, Dallas, TX, USA). The optical intensity threshold was corrected to 0–250 in the process of analyses. The immunohistochemical image acquisition and quantitative analyses were performed by two observers, independently and blinded for the groups. The inter- and intra-examiner reliability was determined. The detailed information of antibodies used in this study is provided in Additional file 2: Table S2.

### Immunofluorescence

Following 10 or 30 days of CCI model construction, the L_4_–L_6_ spinal cord segments were prepared on a cryostat and processed for immunofluorescence as described previously [15]. In brief, the sections were washed twice with PBS, treated with 0.2% Triton X-100 for 20 min, and washed twice with PBS. Then, sections were incubated with 10% goat serum for 2 h at room temperature, and then incubated with the primary antibody against Clec7a,NLRP3,Iba-1,GFAP or NeuN overnight at 4 ℃. In addition, the sections were washed three times with PBS and incubated with secondary antibodies for 1 h atroom temperature. The nuclei were stained with DAPI (S2110, Solarbio) After that, the stained sections were examined via laser scanning confocal microscopy (FluoView FV3000, Olympus Co. Tokyo, Japan). The Clec7a expression was analyzed and expressed as mean fluorescent intensity (integrated option density/area) using the Image-Pro Plus 6.0 (Media Cybernetics, Dallas, TX, USA). In the counting studies of double staining labeled cells (Clec7a/Iba-1, Clec7a/GFAP, Clec7a/NeuN, NLRP3/Iba-1, NLRP3/GFAP and NLRP3/NeuN), the co-labeled cells were counted in 3 representative capture views of the spinal dorsal horn (SDH) from 3 animals in each group. Cell counts on the examined sections were then averaged to provide a single value for the specific group. The detailed information of antibodies used in this study is provided in Additional file 2: Table S2.

### ELISA assay

Rat IL-1β and IL-18 levels were measured by using IL-1β ELISA kit (ml037361, mlbio, Shanghai, China) and IL-18 ELISA kit (ml002816, mlbio, Shanghai, China) according to the manufacturer’s instructions. In brief, samples were measured at 450 nm by spectrophotometer to determine absorbance. The concentrations of IL-1β and IL-18 were obtained by extrapolation from the standard curve.

### LDH assay

Cell death in BV2 cells was assessed using LDH Cytotoxicity Assay Kit (Beyotime, Shanghai, China). The supernatants were collected and centrifuged (400 g, 5 min) for LDH activity determination. The absorbance at 495 nm was measured using a microplate reader.

### TUNEL assay

Pyroptosis in the spinal cord and BV2 cells were detected by using a TUNEL Detection kit (ABP-Biosciences, Wuhan, China). The pyroptotic cells showed TUNEL-positive due to random breakage of chromatin DNA. The samples were incubated with a TdT detection mixture for 1 h at 37 ℃ in the dark, then stained with Hoechst 33,342 (Servicebio, Wuhan, China) for nuclear counterstaining and observed under an inverted microscope. TUNEL-positive nucleus was identified as nicks in the terminal end of nucleic acids. Images were randomly selected from three sections of each specimen. The TUNEL-positive rate was calculated as follows: TUNEL-positive rate % = TUNEL-positive nucleus /total nucleus ×100%.

### Flow cytometry

BV2 cells were washed twice with cold PBS and resuspend cells in 1X Binding Buffer, then stained with FITC Annexin V and PI using the Annexin V-FITC Apoptosis Detection kit (BD, Franklin Lakes, NJ, USA). After incubation at RT in the dark for 15 min, the pyroptotic cells were analyzed by flow cytometry (ACEA Biosciences, Santiago, USA).

### Caspase-1 activity detection

Caspase-1 activity was measured using a commercial Caspase-1 Assay Kit (ab39412, abcam). Briefly, cells were lysed and the lysates were removed by centrifugation at 14,000 rpm/min for 5 min. 100 μg of total protein was used to assess caspase-1 activity. Cell protein with YVAD-AFC were incubated at 37 ℃ for 2 h protected from light. The changes in fluorescence intensity (Ex/m = 400/505 nm) were monitored by a fluorescence spectrophotometer (Varioskan Flash 4FE, Thermo Scientific, Carlsbad, CA, USA).

### Dual-luciferase reporter assay

The 1500-bp sequence upstream of the transcription start site (TSS) of C/EBPβ were amplified and cloned into the effector vector pcDNA3.1-GFP to generate pcDNA3.1-GFP-C/EBPβ. The 2000-bp promoter sequence of Clec7a was truncated in different lengths: p500, p1000, p1500, p2000. Those was respectively amplified and cloned into the reporter vector psiPRO to generate four reporters: psiPRO-Clec7a-: p500, p1000, p1500, p2000. The empty pcDNA3.1-GFP plasmid or psiPRO plasmid was used as a negative control. Based on psiPRO-Clec7a-p500, one mutated reporter was generated, which were mutated at binding site − 483 and − 467. HEK-293 T cells were transfected with these plasmids using Lipofectamine™ 2000 (Invitrogen).After 48 h, the activity of firefly luciferase (FL) and Renilla luciferase (RL) were respectively measured using the Luciferase Assay System protocol (Promega). Relative luciferase activity was represented by FL/RL.

### Chromatin immunoprecipitation (ChIP) assay

ChIP was performed using a Pierce Magnetic ChIP kit (Cat.26157, Thermo Fisher, USA) according to the manufacturers’ instructions. In brief, 1 ×10^7^ cells were used in the experiment. The BV2 cells were cross-linked with 1% paraformaldehyde, and the reaction was stopped with 125 mmol/L glycine. Then, they were lysed with lysis buffer, and chromatin was sonicated (amplitude, 120 w; process time, 8 min; ON time, 3 s; OFF time, 9 s) until crosslinked chromatin was broken into150- to 1000-bp fragments by MNase digestion. The chromatin was then immunoprecipitated with 2 μg (0.6 μg/μL, 3.4 μL) of rabbit anti-C/EBPβ antibody (ab32358, Abcam, Cambridge, MA, USA) or 2 μg of rabbit IgG with rotation overnight at 4 ℃. Then, 20 μL of magnetic beads were added into each tube, and the tubes were incubated overnight at 4 ℃ with mixing. The magnetic beads were washed with IP wash buffer five times and then eluted. After purification, the immunoprecipitated chromatin was analyzed by qRT-PCR. The sequences of primers used in this study are provided in Additional file 2: Table S1.

### Statistical analyses

Data analysis were performed using SPSS 21.0 statistical software (SPSS, Inc, Chicago, IL, USA). Unless otherwise stated, values of n indicated in the Figure Legends refer to the number of independent biological replicates. The data obtained from this study were presented as the mean ± SEM. Significance (*P*-value) was evaluated using either unpaired Student’s t-test or one-way ANOVA followed by Tukey’s multiple comparisons post hoc test or Brown-Forsythe and Welch ANOVA tests followed by a Dunnett's T3 multiple comparisons test for multiple comparisons. Time-series data were analyzed with the two-way ANOVA. *P* < 0.05 was considered to be statistically significant.

## Results

### CCI leads to the upregulation of Clec7a at both mRNA and protein levels in SC

**Fig. 1:**
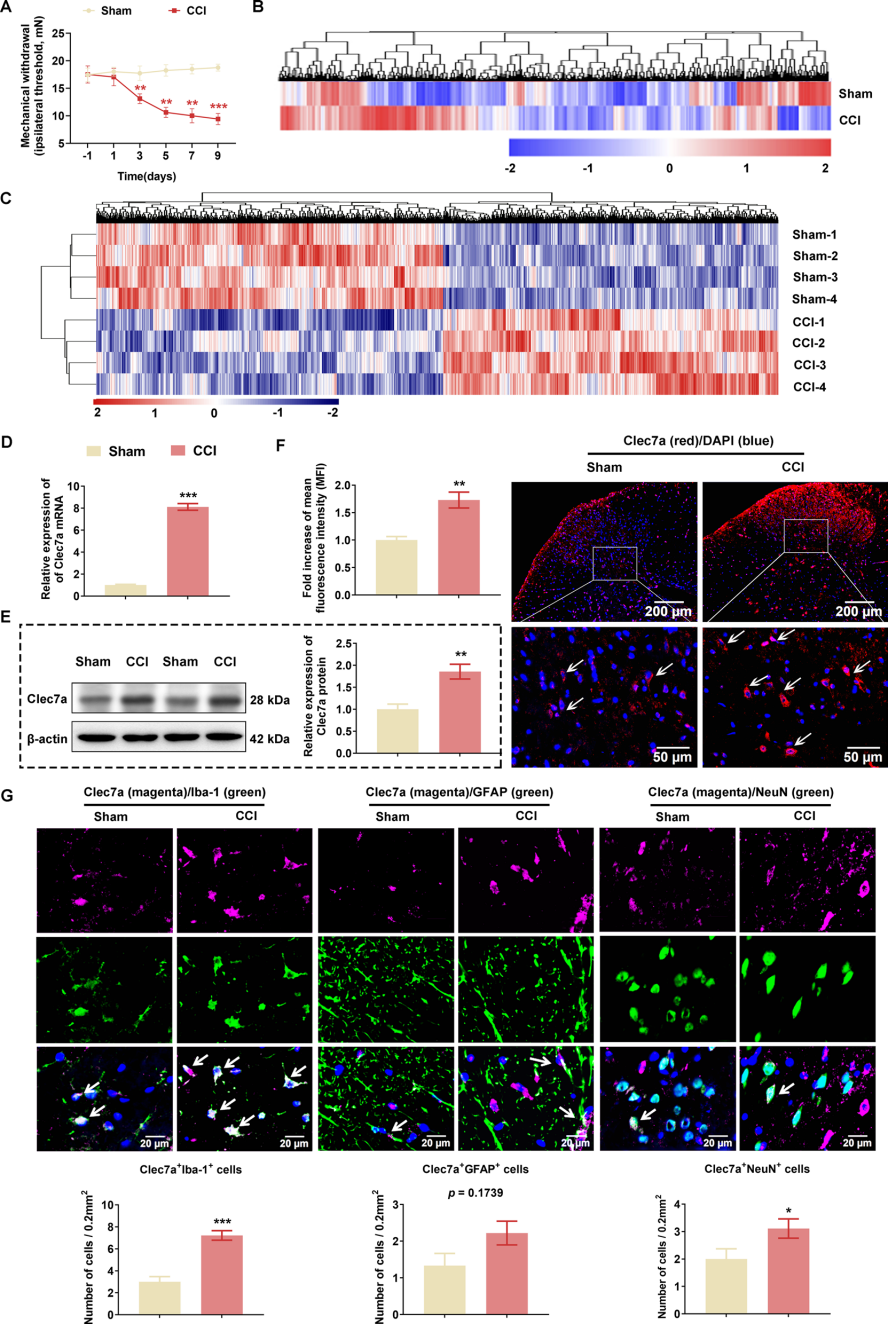
Increased expression of Clec7a in spinal cord of the chronic constriction injury rats. (A) Mechanical withdrawal threshold in the ipsilateral paw of Sham and CCI rats (n = 12 rats/group). (B-C) Hierarchical clustering of differentially expressed genes (DEGs) in spinal cord (SC) between CCI and Sham rats using microarray analyses (B; n = 3 rats/group) and RNA sequencing (C; n = 4 rats/group). (D-E) Expression levels of Clec7a mRNA and protein in SC tissues of Sham and CCI rats determined by qPCR and western blot, respectively (n = 3 rats/group with 2 technical replicates). (F) Clec7a immunofluorescence in the ipsilateral spinal dorsal horns (magnification 5 × /20 ×) on Day 10 (post-nerve injury). White arrows point to Clec7a-positive cells. The mean fluorescence intensity (MFI) was calculated by Image J software (n = 4–6 rats/group, 1 section/rat, 1–2 fields of view/section). Scale bar: 200 μm (up panel), 50 μm (down panel). (G) Representative images and quantitative analysis of co-localization of Iba-1, GFAP or NeuN with Clec7a in the ipsilateral spinal dorsal horns between Sham and CCI rats (n = 3 rats/group, 1 section/rat, 3 fields of view/section). White arrows point to Clec7a-positive cells. Scale bar: 20 μm. Data are presented as mean ± SEM. Unpaired Student’s t test was used to analyze data between the two groups. *P < 0.05, **P < 0.01, ***P < 0.001 compared with Sham group.

**Fig. 2:**
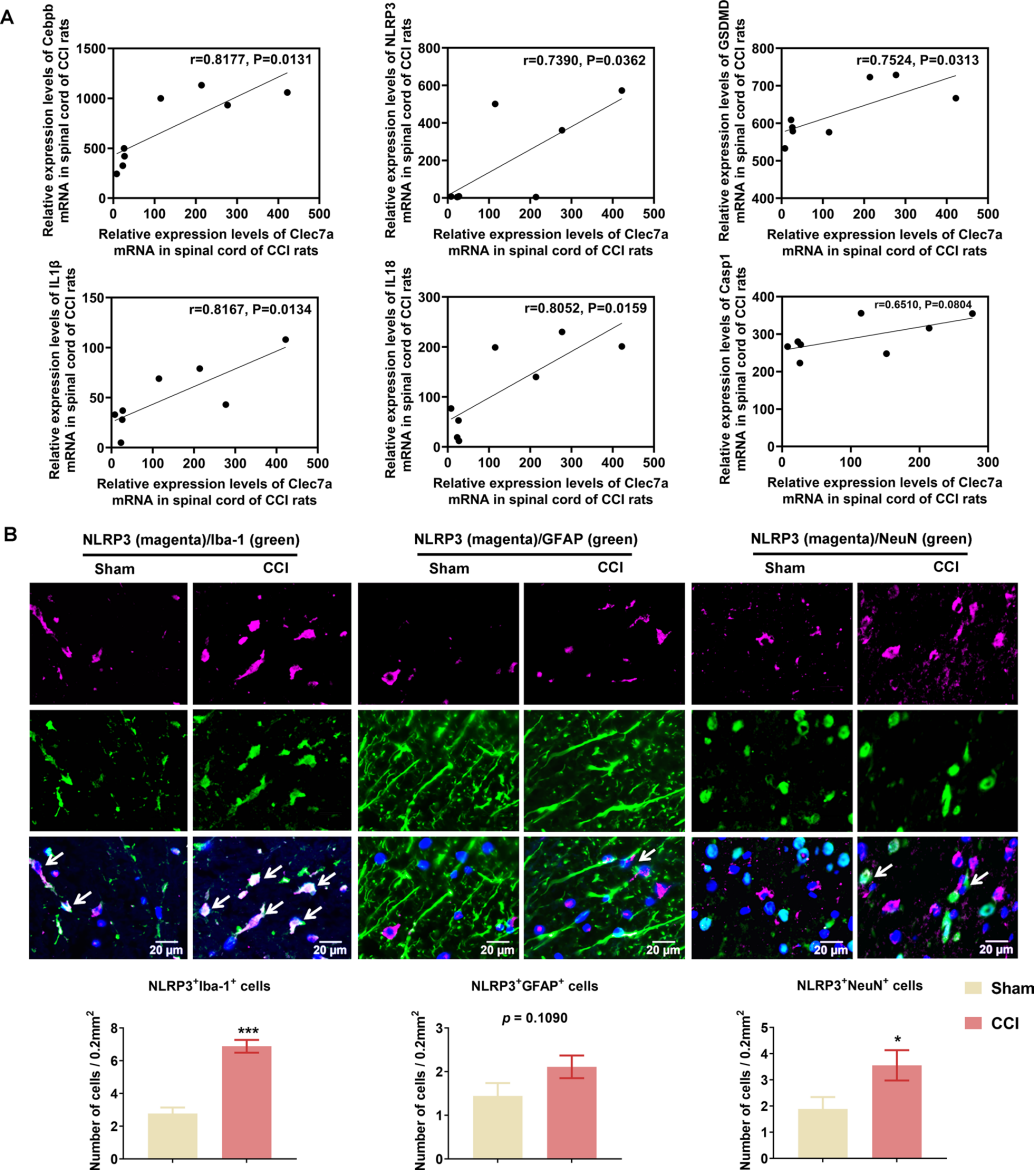
The expression characteristics of molecules involved in the Clec7a-mediated pathway in spinal cord of the chronic constriction injury rats. **A** Pearson correlation analysis between *Clec7a mRNA* and *Cebpb, Nlrp3*, *Gsdmd, Il1b*, *Il18*, *CASP1* expression according to the RNA-seq data (*n* = 4 rats/group). **B** Representative images and quantitative analysis of co-localization of Iba-1, GFAP or NeuN with NLRP3 in the ipsilateral spinal dorsal horns between Sham and CCI rats (*n* = 3 rats/group, 1 section/rat, 3 fields of view/section). White arrows point to Clec7a-positive cells. Scale bar: 20 μm. Data are presented as mean ± SEM. Unpaired Student’s *t* test was used to analyze data between the two groups. *P < 0.05, ***P < 0.001 compared with Sham group

Compared with the Sham rats, MWT was dramatically decreased in the CCI rats starting on the third day, and continued to decline to their lowest levels by the ninth day (Fig. 1A). Both microarray and RNA sequencing studies were performed to determine the gene expression changes during the progression of CCI-induced neuropathic pain. As a result, the two studies respectively identified a total of 602 (GEO: GSE185278, the top 100 DEGs are listed in Additional file 2: Table S3; 62 upregulated genes and 38 downregulated genes) and 1304 (GEO: GSE186237, the top 100 DEGs are listed in Additional file 2: Table S4; 89 upregulated genes and 11 downregulated genes) DEGs between CCI and Sham groups. Then, unsupervised hierarchical clustering of all dysregulated genes showed good differentiation of CCI and Sham groups (Fig. 1B for microarray and Fig. 1C for RNA-seq). Of note, Clec7a was found to be one of the common DEGs in both microarray and RNA-Seq. Accordingly, we verified the marked overexpression of Clec7a at both mRNA (Fig. 1D) and protein (Fig. 1E) levels in SC tissues of CCI rats compared with those of Sham rats on postoperative day 10. Consistently, immunofluorescence staining of SC sections revealed the neuropathic pain-induced signifcant increase of Clec7a expression (Fig. 1F). To further determine which types of cell in the SC tissues mainly expressed Clec7a, the double immunofluorescence staining analysis was performed. Notably, Clec7a was highly colocalized with Iba-1-positive microglia, but less colocalized with NeuN-positive neurons and GFAP-positive astrocytes in the SC tissues of CCI rats (Fig. 1G). In addition, the Pearson correlation analysis based on RNA-seq data showed that the expression of *Clec7a* mRNA was positively correlated with that of *Cebpb, Nlrp3*, *Il1b* and *Il18* mRNAs significantly (Fig. 2A). Moreover, we observed that the cell types positively expressing NLRP3 were the same as that expressing Clec7a (Fig. 2B).

### Suppression of Clec7a relieves neuropathic pain by regulating NLRP3 inflammasome-induced pyroptosis in vivo and in vitro

**Fig. 3:**
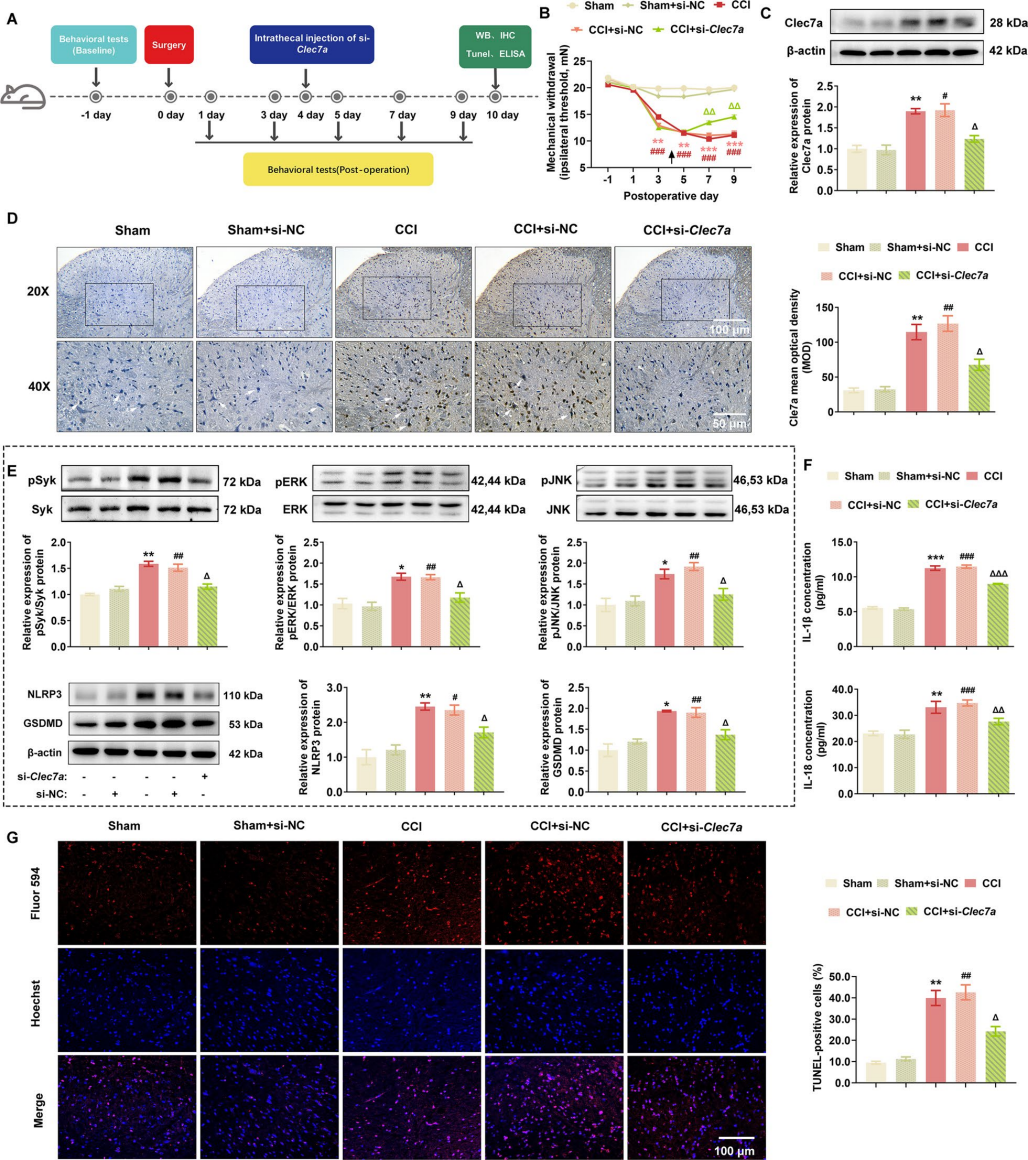
*Clec7a* knockdown relieved neuropathic pain by regulating NLRP3 inflammasome-induced pyroptosis in vivo. **A** Timeline schematic of experimental paradigm. **B** Administration of *Clec7a* siRNA significantly ameliorated mechanical withdrawal threshold in CCI rat model (*n* = 10 rats/group). **C** The upregulation of Clec7a in spinal cord (SC) of CCI rats were significantly suppressed by si-*Clec7a* transfection on postoperative day 10 (*n* = 3 independent blots)*.* **D** Immunohistochemical detection and quantification of Clec7a in ipsilateral SC of CCI rats after si-*Clec7a* treatment (*n* = 6 rats/group, 1 section/rat). White arrows point to Clec7a-positive cells. Scale bar: 100 μm (20 ×), 50 μm (40 ×). **E** Pyroptosis-related proteins (NLRP3, GSDMD) and pSyk, pERK, pJNK in ipsilateral SC of Sham or CCI rats treated with indicated siRNA were evaluated by western blotting (*n* = 3 independent blots). **F** The levels of IL-1β and IL-18 in serum of indicated groups were assessed by ELISA (*n* = 3 rats/group with 3 technical replicates). **G** Representative images (left) and quantification evaluation (right) of Tunel staining in indicated groups (*n* = 5 rats/group, 1 section/rat). Red: Tunel-positive cells; blue: nuclei (Hoechst 33,258). Scale bar: 100 μm. Data are presented as mean ± SEM. Two-way repeated measures ANOVA and Tukey’s post hoc test was used to analyze data at different time points **B**. One-way ANOVA was used to analyze data among multiple groups followed by Tukey’s post hoc test for equal variances or Dunnett T3 post hoc test for unequal variances **C**–**G**. ^*^P < 0.05, ^**^P < 0.01, ^***^P < 0.001 compared with the Sham group; ^#^*P* < 0.05, ^##^*P* < 0.01, ^###^*P* < 0.001 compared with Sham + si-NC group; ^△^*P* < 0.05, ^△△^*P* < 0.01, ^△△△^*P* < 0.001 compared with CCI + si-NC group

To determine the involvement of Clec7a in neuropathic pain progression, *Clec7a*-specific siRNA was injected into CCI rats on postoperative day 4 (Fig. 3A), and the MWT data showed that si-*Clec7a* markedly attenuated the progression of mechanical allodynia (Fig. 3B). Consistently, CCI-induced elevation of Clec7a protein was significantly decreased by si-*Clec7a* in the spinal dorsal horn ipsilateral to the CCI (P = 0.015 and 0.002, Fig. 3C, D). Regarding to the correlation between pyroptosis and Clec7a in CCI-induced neuropathic pain, our western blot data showed that Clec7a-triggered phosphorylation of Syk, ERK and JNK, as well as pyroptosis-related proteins NLRP3 and GSDMD were markedly upregulated in spinal dorsal horn ipsilateral to the CCI, which were significantly reversed by si-*Clec7a* intrathecal injection **(**Fig. 3E**)**. Accordingly, the knockdown of Clec7a expression by the specific siRNA effectively reduced the release of pyroptosis related inflammatory cytokines (IL-1β and IL-18) in the serum (Fig. 3F) and the percentage of Tunel-positive cells in spinal dorsal horn tissues (Fig. 3G).

After that, Clec7a siRNA was applied for the LPS combined with ATP-treated BV2 cells. As shown in Additional file 1: Figure S1A. LPS combined with ATP treatment led to an increasing number of BV2 cells which exhibited pyroptosis-like morphological changes, such as increased cell size, swelling and bubble-like protrusions on cytomembrane, while Clec7a siRNA transfection dramatically reduced the extent of pyroptosis. Similarly, flow cytometric results also showed that the LPS combined with ATP-induced pyroptosis cells were significantly decreased by Clec7a siRNA transfection (all P < 0.05, Additional file 1: Figure S1B). Moreover, the expression levels of Clec7a, NLRP3, GSDMD and cleaved-GSDMD proteins were significantly decreased following Clec7a siRNA transfection (all P < 0.05, Additional file 1: Figure S1C). The pyroptosis related activation of caspase-1, release of lactic dehydrogenase (LDH) and inflammation cytokines (IL-1β and IL-18) in the cell lysis or culture supernatants were also remarkably reduced in the BV2 cells with the knockdown of Clec7a (all P < 0.05, Additional file 2: Figure S1D–F), in line with the observation in the Tunel staining (all P < 0.05, Additional file 1: Figure S1G).

These data suggest that Clec7a may play an essential role in the progression of neuropathic pain, and the knockdown of Clec7a may attenuate neuropathic pain by regulating NLRP3 inflammasome-dependent pyroptosis.

### Clec7a may be a target gene of transcription factor C/EBPβ

**Fig. 4:**
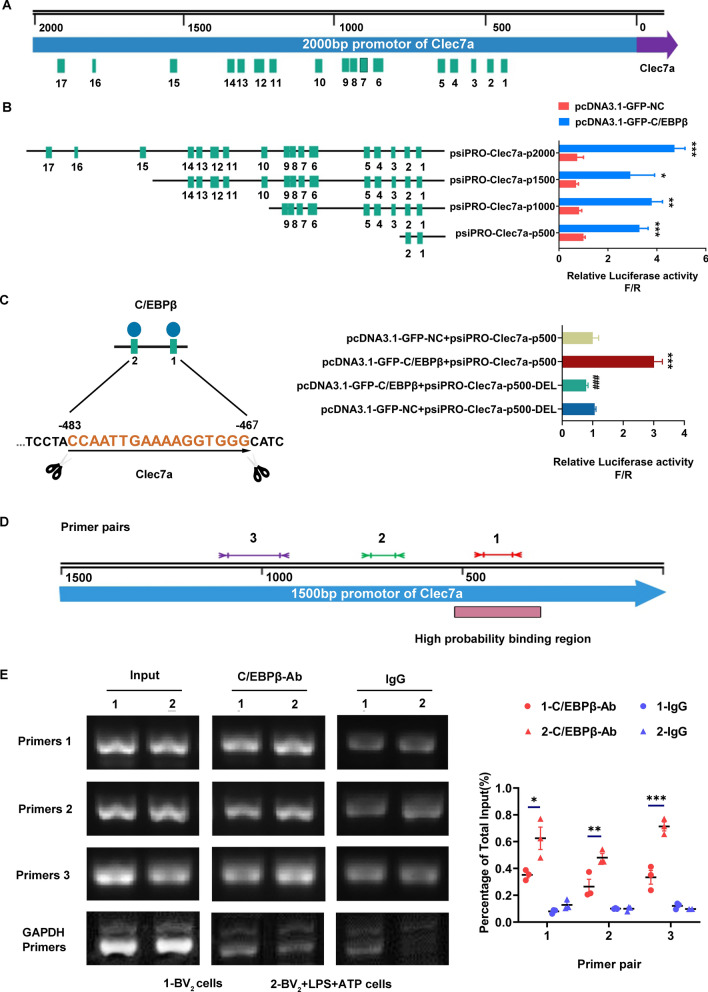
C/EBPβ is a transcription activator in the promoter region of *Clec7a* gene. (**A** and **B**) Schematic representation of a series of 5’ unidirectional deletions of the 2000 bp *Clec7a* promoter region fused in frame to pcDNA3.1 luciferase reporter vector, and then the promoter activity of C/EBPβ was determined by measuring the fluorescence intensity. C/EBPβ binding elements in the promoter region of *Clec7a* were predicted using the transcription activator search software, and shown as cyan boxes (*n* = 3). ^*^P < 0.05, ^**^P < 0.01, ^***^P < 0.001 compared with pcDNA3.1-GFP-NC group via unpaired Student’s *t* test. (**C**) Mutated C/EBPβ binding sites on *Clec7a* promoter-driven luciferase reporters were constructed, and then the luciferase activity was analyzed via one-way ANOVA followed by Tukey’s post hoc test (*n* = 3). ^***^P < 0.001 compared with the pcDNA3.1-GFP-NC + psiPRO-Clec7a-p500 group. ^###^*P* < 0.001 compared with the pcDNA3.1-GFP-NC + psiPRO-Clec7a-p500-DEL group. **D** The location of amplified fragments of three pairs of primers on *Clec7a* promoter region. **E** Chromatin immunoprecipitation (ChIP) analysis of C/EBPβ binding at the *Clec7a* promoter. The anti-C/EBPβ antibody or IgG control was used to pull down the DNA–protein complex from the control or LPS + ATP treated BV2 cells as indicated. Subsequently, immunoprecipitated DNA was determined by PCR and quantitative real-time PCR with three pairs of primers (*n* = 3). Data are presented as mean ± SEM. One-way ANOVA followed by Tukey’s post hoc test was used to analyze data among multiple groups. ^*^P < 0.05, ^**^P < 0.01, ^***^P < 0.001 compared with the BV2-C/EBPβ antibody group

To search for putative transcription factor binding motifs of *Clec7a*, in silico computational analysis using TFSEARCH Search software (http://www.cbrc.jp/research/db/TFSEARCH.html) was employed to locate predicted binding motifs [16]. As shown in Fig. 4a total of 17 potential C/EBPβ regulatory elements were predicted to be within the promoter region (proximal 2000 bp 5’ flanking region) of *Clec7a.* To further characterize the potential C/EBPβ binding motifs on the *Clec7a* promoter, four progressive deletion reporter constructs of the 5’ flanking region were generated and transfected in HEK293T cells, and then luciferase activity was analyzed. Firefly luciferase expression was activated if the Clec7a vector upstream of luciferase contained the C/EBPβ binding motifs, then luciferase catalyzed the substrate luciferin and emit bioluminescence. Renilla luciferase, which used coelenterate as the substrate, was driven by a fixed constitutive promoter and served as an internal reference signal for correcting input errors in the reporting system.The activity of each reporter construct was directly measured by the ratio of firefly luciferase activity to Renilla luciferase activity (F/R). Among all promoter constructs, the deletion of distal 5’ C/EBPβ binding motifs (500–2000 bp) did not affect the transcriptional activity as compared to the reporter construct containing the first two C/EBPβ binding motifs (0–500 bp) (Fig. 4B). After that, the mutanted C/EBPβ binding sites (binding motif 1, 2) on *Clec7a* promoter-driven luciferase reporters were constructed, and the transcriptional activity of the mutanted C/EBPβ binding sites constructs decreased by 3.5-fold, indicating that C/EBPβ may be an certain promoter of *Clec7a* (P < 0.001, Fig. 4C). To verify C/EBPβ binding to the endogenous *Clec7a* promoter region, CHIP assay was employed using anti-C/EBPβ antibody or IgG control to pull down the DNA–protein complex from the control or LPS combined with ATP treated BV2 cells. Subsequently, immunoprecipitated DNA was determined by qPCR with three pairs of primers (Fig. 4D). qPCR data revealed that the target gene (*Clec7a*) of C/EBPβ was remarkably increased following LPS combined with ATP incubation (Fig. 4E), suggesting that C/EBPβ may bind to the *Clec7a* promoter region and activate *Clec7a* transcription.

### Disruption of the C/EBPβ-Clec7a axis relieves neuropathic pain by regulating NLRP3 inflammasome-induced pyroptosis in vivo and in vitro

**Fig. 5:**
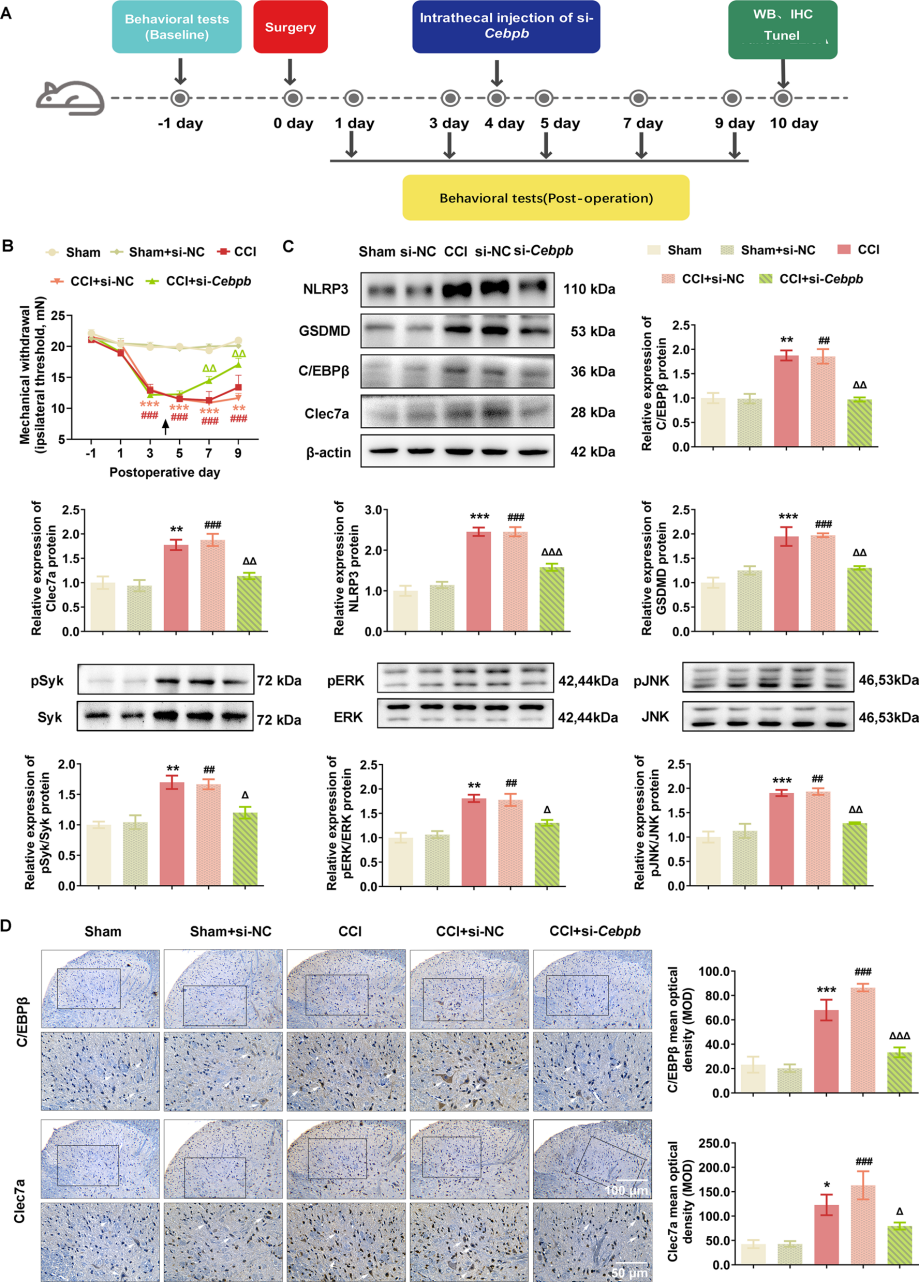
C/EBPβ knockdown attenuated neuropathic pain by regulating Clec7a-NLRP3 induced pyroptosis in vivo. **A** Timeline schematic of experimental paradigm. **B** The decreased expression of C/EBPβ significantly ameliorated mechanical withdrawal threshold in CCI rat model (*n* = 5–10 rats/group). **C** Intrathecally administration of *Cebpb* siRNA significantly reduced the expression of C/EBPβ, Clec7a, pyroptosis-related proteins (NLRP3, GSDMD) and pSyk, pERK, pJNK in ipsilateral spinal cord of CCI rats on postoperative day 10 (*n* = 3 independent blots). **D** Immunohistochemical detection of C/EBPβ and Clec7a in ipsilateral SC of CCI rats after si-*Cebpb* administration (*n* = 6 rats/group, 1 section/rat). White arrows point to C/EBPβ- or Clec7a-positive cells, respectively. Scale bar: 100 μm (20 ×); 50 μm (40 ×). Data are presented as mean ± SEM. Two-way repeated measures ANOVA and Tukey’s post hoc test was used to analyze data at different time points **B**. One-way ANOVA was used to analyze data among multiple groups followed by Tukey’s post hoc test for equal variances or Dunnett T3 post hoc test for unequal variances **C**–**D**. ^*^P < 0.05, ^**^P < 0.01, ^***^P < 0.001 compared with the Sham group; ^##^*P* < 0.01, ^###^*P* < 0.001 compared with sham + si-NC group; ^△^*P* < 0.05, ^△△^*P* < 0.01, ^△△△^*P* < 0.001 compared with CCI + si-NC group

**Fig. 6:**
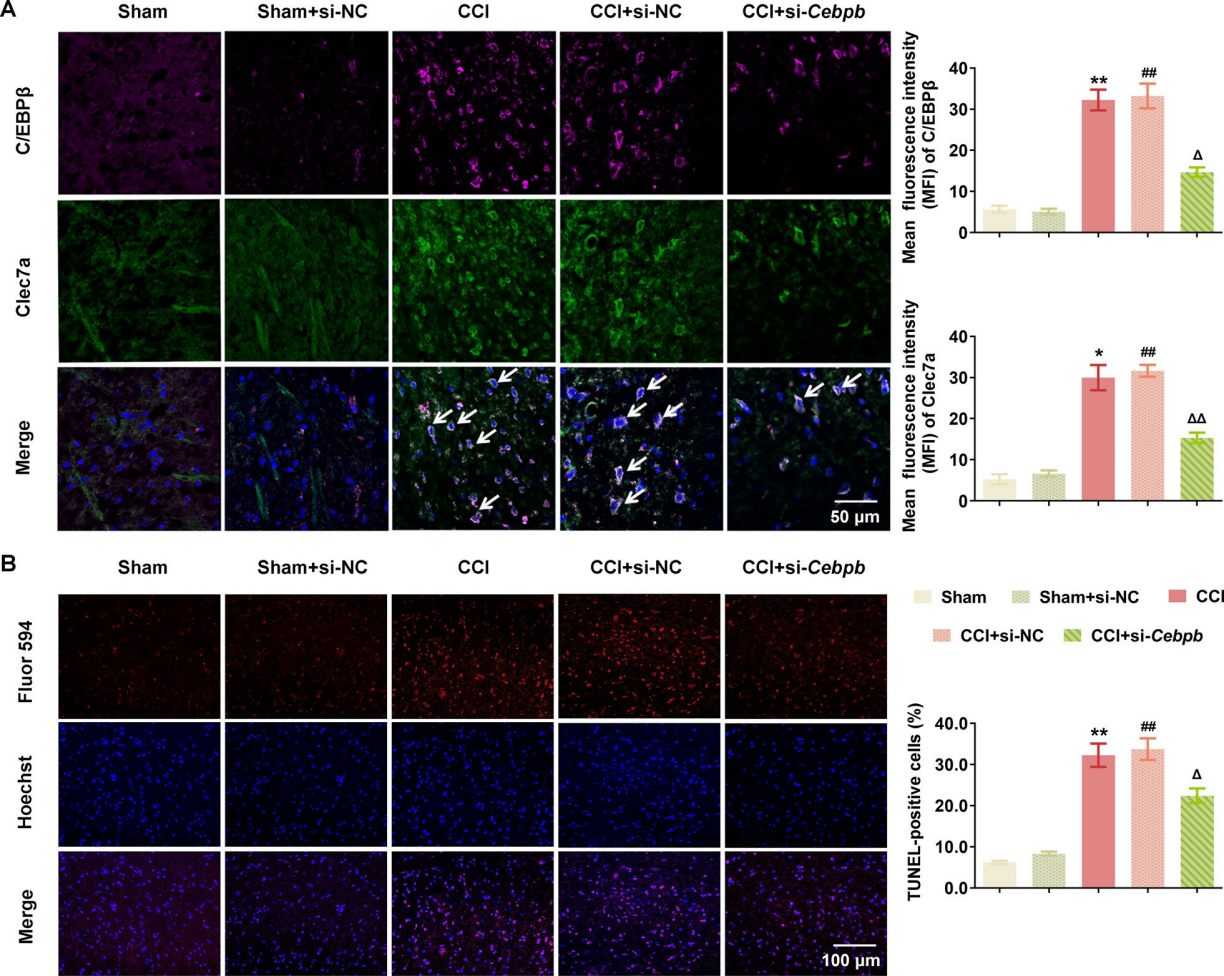
C/EBPβ knockdown inhibited the overexpression of Clec7a and NLRP3 induced pyroptosis in spinal cord of the chronic constriction injury rats. **A** The double immunofluorescence staining of C/EBPβ and Clec7a in ipsilateral SC of CCI rats after si-Cebpb administration (n = 6 rats/group, 1 section/rat). White arrows point to C/EBPβ- and Clec7a-positive cells. Scale bar: 50 μm. **B** Representative images (left) and quantification evaluation (right) of Tunel staining in the indicated groups (*n* = 6 rats/group, 1 section/rat). Red: Tunel-positive cells; blue: nuclei (Hoechst 33,258). Scale bar: 100 μm. Data are presented as mean ± SEM. One-way ANOVA was used to analyze data among multiple groups followed by Tukey’s post hoc test for equal variances or Dunnett T3 post hoc test for unequal variances. ^*^P < 0.05, ^**^P < 0.01 compared with the Sham group; ^##^*P* < 0.01 compared with sham + si-NC group; ^△^*P* < 0.05, ^△△^*P* < 0.01 compared with CCI + si-NC group

The above data prompted us to investigate the involvement of C/EBPβ-Clec7a axis into neuropathic pain progression. *Cebpb*-specific siRNA (2 μg, i.t.) was injected into CCI rats on postoperative day 4 (Fig. 5A), and the MWT data showed that si-*Cebpb* markedly attenuated the progression of mechanical allodynia (Fig. 5B). Consistently, immunoblotting, IHC and IF data showed that CCI-induced elevation of C/EBPβ and Clec7a protein were remarkably decreased by si-*Cebpb* intrathecal injection in the spinal dorsal horn ipsilateral of CCI rats (Figs. 5C, D and 6A). Moreover, the double immunofluorescence staining analysis demonstrated that Clec7a was highly colocalized with C/EBPβ in the SC tissues of CCI rats (Fig. 6A). In addition, western blot analysis demonstrated that Clec7a-triggered phosphorylation of Syk, ERK and JNK, as well as the pyroptosis-related proteins NLRP3 and GSDMD were markedly upregulated in spinal dorsal horn ipsilateral of CCI rats (Fig. 5C), while the pyroptosis was significantly attenuated by si-*Cebpb* intrathecal injection, in line with which, the percentage of Tunel-positive cells in dorsal horn was dramatically elevated after CCI modeling, and intrathecal injection of si-*Cebpb* reduced the Tunel-positive cells of CCI rats (P = 0.001, Fig. 6B).

To explore the effects of C/EBPβ-Clec7a axis on pyroptosis in vitro, C/EBPβ knockdown BV2 cell line was constructed using lentivirus transfection system. As shown in Additional file 1: Figure S2A, LPS combined with ATP treatment led to the pyroptosis-like morphological changes in a large number of BV2 cells, while BV2-pLV.sh-*Cebpb* cells exhibited resistance to the combination of LPS and ATP induced pyroptosis, which was consistent with the flow cytometric data (P < 0.001, Additional file 1: Figure S2B). Moreover, the expression of C/EBPβ, Clec7a, NLRP3, GSDMD, and cleaved-GSDMD proteins in BV2-pLV.sh*Cebpb* cells were remarkably lower than that in the BV2-pLV.shCtrl cells (Additional file 1: Figure S2C). The caspase-1 activity, release of LDH and inflammation cytokines (IL-1β and IL-18) in the cell lysis or culture supernatants were also elevated by the LPS combined with ATP treatment, which was significantly reduced by the knockdown of C/EBPβ (Additional file 1: Figure S2D-F), in line with the observation of the Tunel staining (P = 0.004, Additional file 1: Figure S2G).

**Fig. 7:**
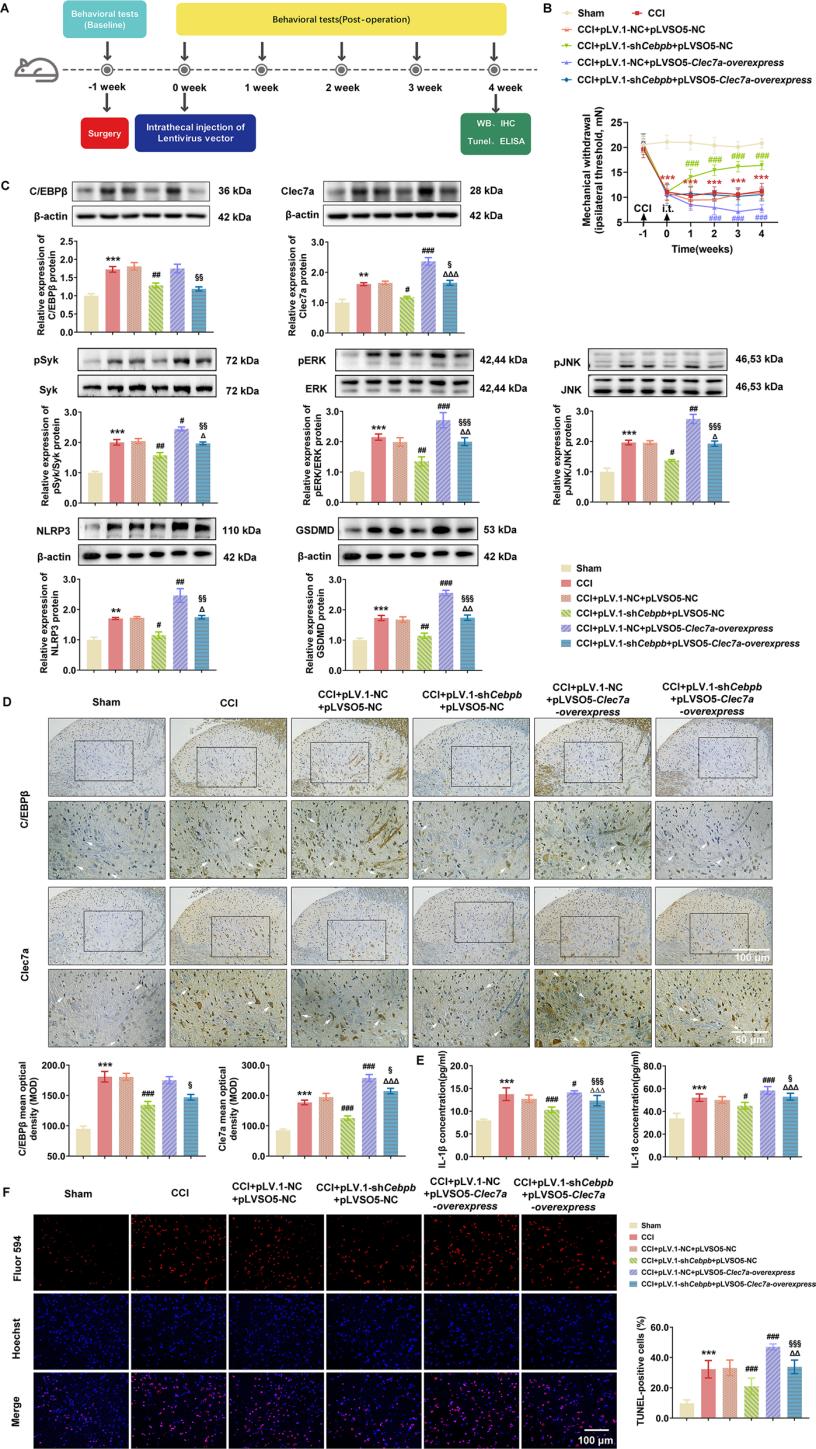
Disruption of the C/EBPβ-Clec7a axis attenuated neuropathic pain by regulating NLRP3 inflammasome-induced pyroptosis in vivo. **A** Timeline schematic of experimental paradigm. **B** The decreased expression of C/EBPβ significantly ameliorated mechanical withdrawal threshold in CCI rat model, while overexpression Clec7a facilitated neuropathic pain (Sham group, *n* = 9; the other groups, *n* = 15 rats/group). **C** Expression levels of C/EBPβ, Clec7a, pSyk, pERK, pJNK, NLRP3 and GSDMD proteins in ipsilateral spinal cord (SC) of CCI rats on postoperative 4 weeks were measured using western blot analysis (*n* = 3 independent blots). **D** Immunohistochemical detection of C/EBPβ and Clec7a in ipsilateral SC of CCI rats after treatment (*n* = 9 rats/group, 1 section/rat). White arrows point to C/EBPβ- or Clec7a-positive cells, respectively. Scale bar: 100 μm (20 ×); 50 μm (40 ×). **E** The concentration of IL-1β and IL-18 in serum of indicated groups were assessed by ELISA (*n* = 3 rats/group with 3 technical replicates). **F** Representative images (up) and quantification evaluation (down) of Tunel staining in the indicated groups (*n* = 6 rats/group, 3 section/rat). Red: Tunel-positive cells; blue: nuclei (Hoechst 33,258). Scale bar: 100 μm. Data are presented as mean ± SEM. Two-way repeated measures ANOVA and Tukey’s post hoc test was used to analyze data at different time points **B**. One-way ANOVA was used to analyze data among multiple groups followed by Tukey’s post hoc test for equal variances or Dunnett T3 post hoc test for unequal variances **C**–**F**. ^**^P < 0.01, ^***^P < 0.001 compared with the Sham group; ^#^*P* < 0.05, ^##^*P* < 0.01, ^###^*P* < 0.001 compared with CCI + pLV.1-NC + pLVSO5-NC group; ^△^*P* < 0.05, ^△△^*P* < 0.01, ^△△△^*P* < 0.001 compared with CCI + pLV.1-sh*Cebpb* + pLVSO5-NC group; ^§^*P* < 0.05, ^§§^*P* < 0.01, ^§§§^*P* < 0.001 compared with CCI + pLV.1-NC + pLVSO5- *Clec7a-*overexpress group

To further determine the effect of C/EBPβ-Clec7a on neuropathic pain and exclude the off-target effect of RNA interference experiment involves C/EBPβ and Clec7a knockdown, the 5 μl of concentrated lentivirus-transduced sh-*Cebpb,* overexpressing-*Clec7a* and/or vector alone without insertion (1 ×10^9^ transducing units/mL) were injected into CCI rats on postoperative day 7 to knockdown C/EBPβ and/or enforce Clec7a expression (Fig. 7A). As shown in Fig. 7B**,** the MWT data showed that pLV.1-*Cebpb* markedly attenuated the progression of mechanical allodynia in the next 4 weeks (P < 0.001), while overexpressing Clec7a by pLVSO5-*Clec7a* exacerbated the allodynia of CCI rats (P < 0.001). Western blot analysis and IHC showed that CCI-induced elevation of C/EBPβ and Clec7a proteins were remarkably decreased by pLV.1-*Cebpb* intrathecal injection in the spinal dorsal horn ipsilateral of CCI rats (Fig. 7C and D), while pLVSO5-*Clec7a* effectively recovered the expression of Clec7a. Furthermore, C/EBPβ knockdown significantly reduced the expression levels of NLRP3, GSDMD, IL-1β and IL-18 proteins, and suppressed the phosphorylated Syk, ERK and JNK levels, which were subsequently reversed by the enforced expression of Clec7a (Fig. 7C, E). Consistent with the western blot data, the percentage of Tunel-positive cells in dorsal horn was dramatically decreased after pLV.1-sh*Cebpb* injection compared to CCI rats (P < 0.001), while pLVSO5-Clec7a injection elevated the Tunel-positive cells compared to the CCI rats injected with pLV.1-sh*Cebpb* (P < 0.001, Fig. 7F).

## Discussion

**Fig. 8:**
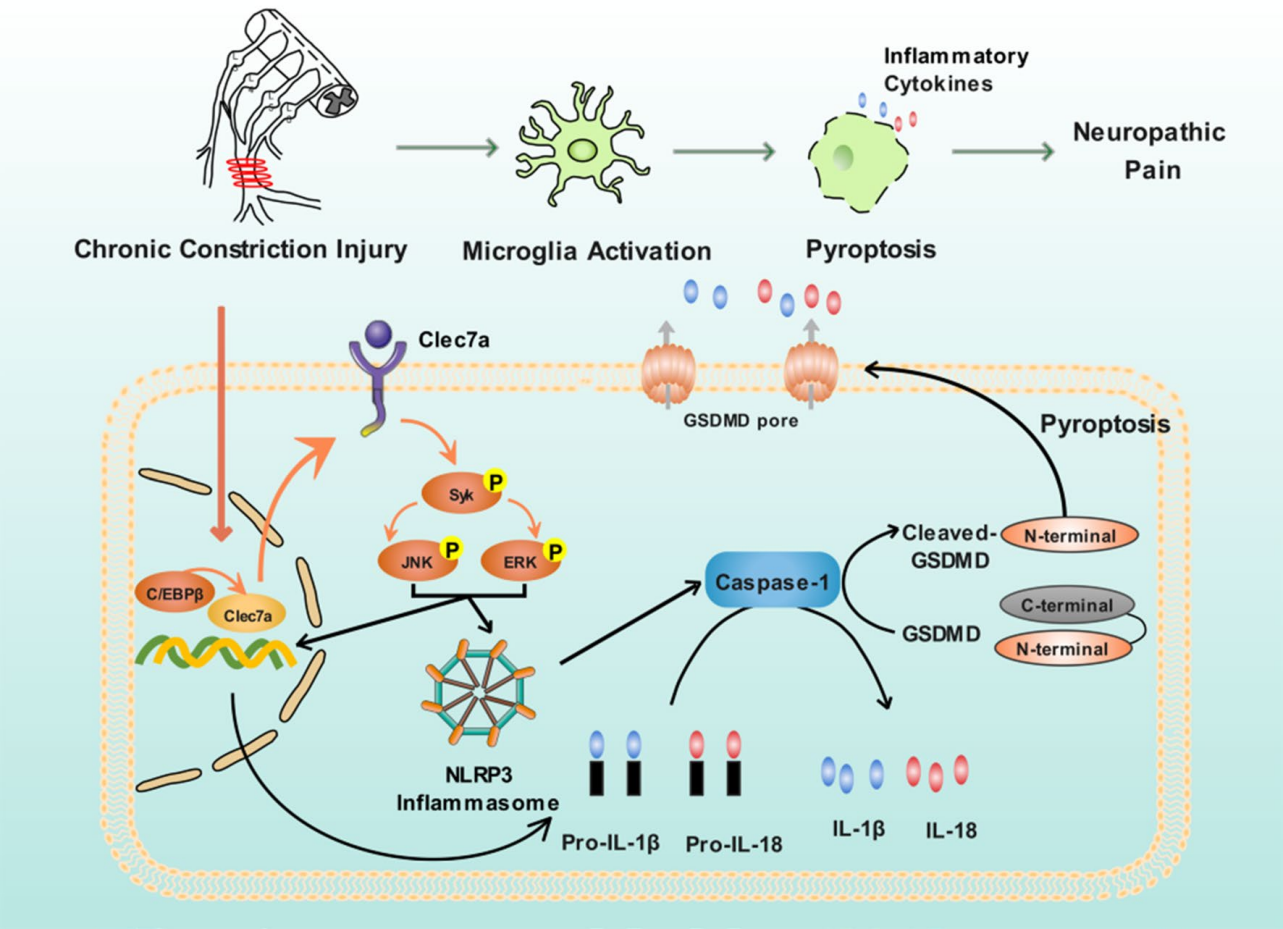
Illustration on the critical role of C/EBPβ-Clec7a axis in neuropathic pain progression, which supports the idea that CCI-induced overexpression of Clec7a, and the direct interaction between C/EBPβ and Clec7a observed along the pain pathways may contribute to neuropathic pain by triggering the phosphorylation of Syk, ERK and JNK proteins, as well as activating NLRP3 inflammasome-mediated pyroptosis

Growing evidence reveal that inflammatory responses play crucial roles in the damage of neuropathic pain, and the pharmaceutical suppression on inflammatory responses may reduce neuronal injury, which subsequently leads to the attenuation in neuropathic pain-induced behaviors [17–19]. Therefore, the clarification on the involvement of inflammatory responses on neuropathic pain is of great significance to identify novel and efficient therapeutic targets for the treatment of this disease. Herein, the current study reveals a novel and critical role of C/EBPβ-Clec7a axis in neuropathic pain progression, which supports the idea that CCI-induced overexpression of Clec7a, and the direct interaction between C/EBPβ and Clec7a observed along the pain pathways may contribute to neuropathic pain by triggering the phosphorylation of Syk, ERK and JNK proteins, as well as activating NLRP3 inflammasome-mediated pyroptosis (Fig. 8). Our principal findings are as follows: a) The spinal cord injury (via CCI treatment) causes the upregulation of Clec7a mRNA and protein in the rat SC; b) The knockdown of Clec7a prevents and suppresses the production and persistence of CCI-induced neuropathic pain via mechanical hypersensitivity; c) Clec7a may be a target gene of transcription factor C/EBPβ, which may enhance the expression of Clec7a in SC induced by CCI; d) C/EBPβ-Clec7a axis may be a potential therapeutic target of the administration of neuropathic pain, evidenced by its relationship with NLRP3 inflammasome-dependent pyroptosis and the contribution to neuroinflammation.

Clec7a, also known as Dectin-1, is a transmembrane receptor protein for β-1,3 and β-1,6 glucans, which is mainly expressed on macrophages, monocytes, microglia, neutrophils, most subsets of dendritic cells, and subpopulations of T-cells, B-cells, mast cells, and eosinophils [20]. As a pattern-recognition receptor, Clec7a has been found to recognize a broad range of microbial pathogens, including fungi and bacteria, which can cause autoimmune disorders and cancer [21]. It also plays a key role in regulating neuroinflammation in ischemic stroke, spinal cord injury and intracerebral hemorrhage [22–24]. A previous study of Rojewska group firstly observed the upregulation of *Clec7a* mRNA in both DRG and SC seven days after CCI-induced neuropathic pain using microarray and qPCR analyses [25], in line with our findings. Fu and colleagues indicated that the inhibition of Clec7a could ameliorate neuroinflammation after intracerebral hemorrhage in mice [21]; Deerhake and others also reported that Clec7a could limit autoimmune neuroinflammation in mice [26]. Consistently, our data demonstrated that intrathecal siRNA-mediated suppression of *Clec7a* effectively attenuated CCI-induced mechanical allodynia, implying a crucial role of *Clec7a* during neuropathic pain progression, which prompt us to investigate its underlying mechanisms.

Inflammatory responses are initiated by pattern recognition receptors, such as Clec7a, which may trigger Syk-JNK signal pathway to transfer cellular danger signals to inflammasomes, such as the NLRP3 inflammasome, resulting in the induction of pro-inflammatory cytokines, such as IL-1β and IL-18, or activation of caspase-1, leading to inflammation or GSDMD-mediated pyroptosis [27–30]. In this study, we revealed a novel role of Clec7a in promoting pyroptosis of CCI mice and BV2 cells, as well as its underlying mechanisms. We found that the knockdown of Clec7a significantly impaired LPS combined with ATP-induced neuroinflammation, Syk/JNK phosphorylation levels and NLRP3 inflammasome-associated molecule expression, suggesting that Clec7a may play crucial roles in the inflammation and pyroptosis of microglia cells. To further explore the upstream molecular mechanisms that influence Clec7a expression, we also investigated transcription factors that may regulate Clec7a. C/EBPβ, as a pleiotropic transcription factor, is an important member of the C/EBP family, and functionally regulates the expression levels of various genes. Emerging evidence has been reported that C/EBPβ participates in the occurrence of inflammation-related diseases via controling multiple genes' expression implicated in immune and inflammatory responses [31, 32]. However, there is no report regarding to the effect of C/EBPβ on Clec7a during pyroptosis. In this study, we confirmed that C/EBPβ directly targeted the promoter region of Clec7a to promoting its expression, and the positive correlation between C/EBPβ and Clec7a expression levels in CCI mice. In addition, the knockdown of C/EBPβ effectively inhibited the release of inflammatory cytokines and pyroptosis by down-regulating Clec7a in CCI mice and BV2 cells.

Notably, NLRP3 inflammasome-regulated inflammatory response has been revealed to be involved into the neuropathic pain occurrence and development [33–35]. However, the relationships between neuropathic pain and pyroptosis have not been fully elucidated. In this study, we found that the knockdown of Clec7a or C/EBPβ markedly attenuated LPS combined with ATP-triggered inflammatory response and the expression of NLRP3, GSDMD-N, caspase-1, IL-1β and IL-18, which suggested that C/EBPβ-Clec7a axis positively regulates LPS combined with ATP-induced pyroptosis by activating NLRP3-caspase-1 dependent inflammasome in rat microglia.

In conclusion, the current pre-clinical investigation reveals that C/EBPβ-Clec7a axis may be a potential target for relieving neuropathic pain through alleviating neuroinflammation, paving its way for clinical translation as a promising approach for neuropathic pain therapy.

## Supplementary Information

**Additional file 1. Figure S1.** *Clec7a* knockdown alleviated LPS and ATP induced BV2 cells pyroptosis. **Figure S2.** C/EBP β knockdown alleviated LPS and ATP induced BV2 cells pyroptosis.

**Additional file 2.  Table S1.** The information of siRNA and primer sequences used in this study. **Table S2.** The information of antibodies used in this study. **Table S3.** E xpression patterns of 100 di fferentially expressed genes in SC between the sham and CCI model groups by Microarray Profiling (mixed sample, n = 3). **Table S4.** Expression patterns of 100 differentially expressed genes in SC between the sham and CCI model groups by RNA-seq (n = 4).

## Data Availability

The datasets generated and analysed during the current study are available in the GEO datasets (https://www.ncbi.nlm.nih.gov/geo/query/acc.cgi?acc=GSE186237 and https://www.ncbi.nlm.nih.gov/geo/query/acc.cgi?acc=GSE185278).

## Abbreviations

Clec7a: C-Type lectin domain containing 7A
CCI: Chronic constriction injury
caspase-1: Cysteine-aspartic protease 1
IL-1β: Interleukin-1β
NLRP3: NOD-like receptor family pyrin domain-3
RNA-seq: RNA sequencing
SC: Spinal cord
siRNA: RNA interference
ELISA: Enzyme-linked immunosorbent assay
IASP: International association for the study of pain
MWT: Mechanical withdrawal threshold
DEGs: Differentially expressed genes
si-Clec7a: Clec7a siRNA
si-Cebpb: C/EBPβ siRNA
si-NC: Control siRNAs
shCebpb: ShRNAs targeting C/EBPβ
shNC: ShRNAs targeting negative control
PBS: Phosphate-buffered saline
FL: Firefly luciferase
RL: Renilla luciferase
ChIP: Chromatin immunoprecipitation
