# Supplementary material for: Disruption of C/EBPβ-Clec7a axis exacerbates neuroinflammatory injury via NLRP3 inflammasome-mediated pyroptosis in experimental neuropathic pain
Source: J Transl Med. 2022 Dec 12;20:583. doi: 10.1186/s12967-022-03779-9 (PMC9743596; doi:10.1186/s12967-022-03779-9)
Supplement: Supplementary file 1 — Additional file 1. Figure S1. Clec7a knockdown alleviated LPS and ATP induced BV2 cells pyroptosis. Figure S2. C/EBP β knockdown alleviated LPS and ATP induced BV2 cells pyroptosis. [file 12967_2022_3779_MOESM1_ESM.pdf]

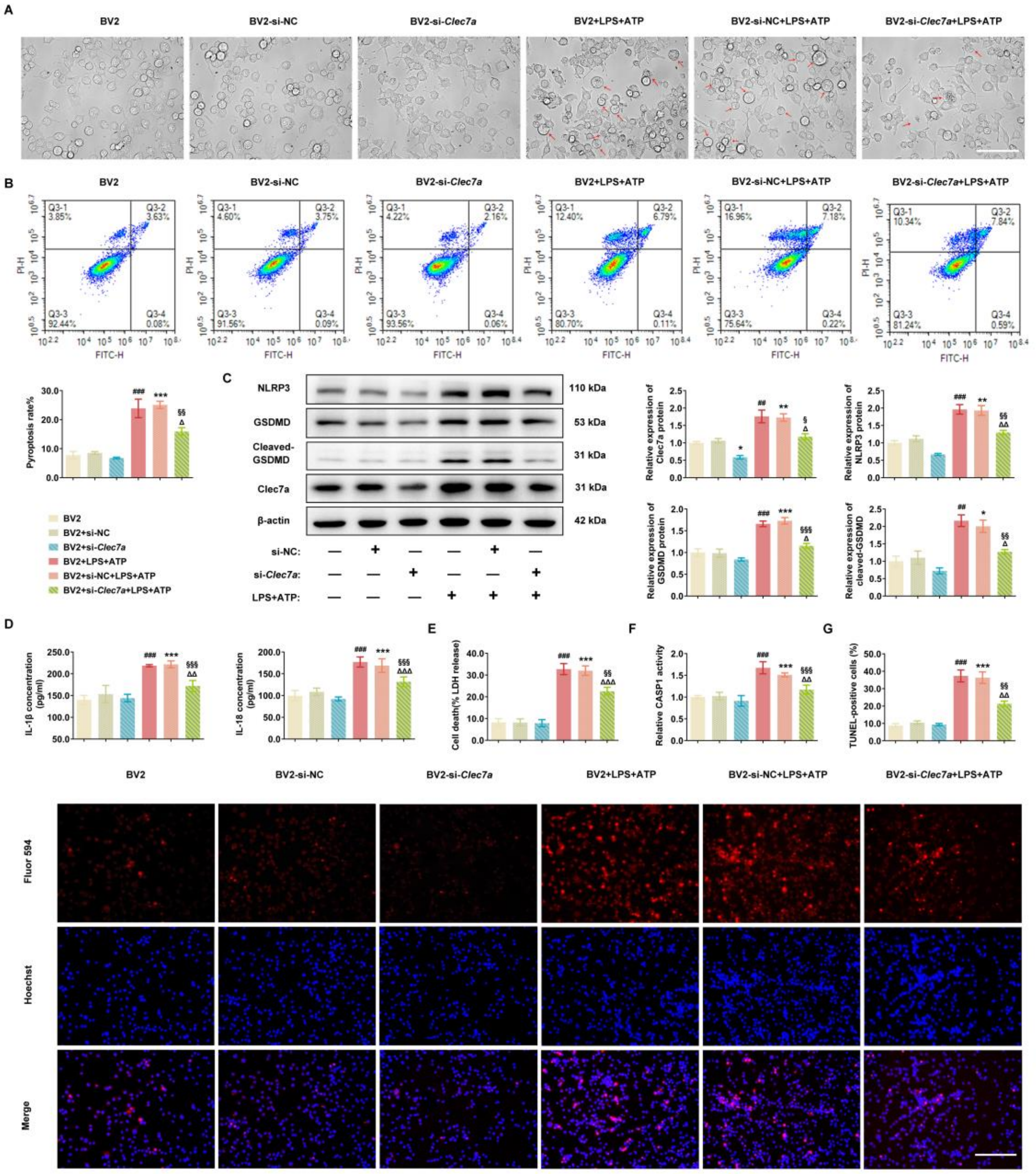

Additional file 1: Figure S1. ***Clec7a* knockdown alleviated LPS and ATP induced BV2 cells pyroptosis.** (A) Representative images of BV2 cells with or without siRNA transfection after the stimulation of LPS+ATP. Arrows point to the pyroptosis-like morphological changes. Scale bar: 500 μm. (B) Pyroptosis was assessed by annexin V-FITC/PI assay after incubated with LPS+ATP in indicated groups (up: representative flow cytometric dotplots; down: quantification of annexin V-FITC and PI

double stained cells) (*n* = 3 wells/group with 3 technical replicates). (C) Pyroptosis related proteins in cells treated with indicated components were evaluated by western blotting (*n* = 3 independent blots). (D) The concentration of IL-1β and IL-18 in culture supernatants were assessed by ELISA (*n* = 3 wells/group with 3 technical replicates). (E) LDH release in the culture supernatants of indicated groups were determined by colorimetry (*n* = 3 wells/group with 3 technical replicates). (F) Caspase-1 activity in the cell lysis of indicated groups were determined by colorimetry (*n* = 3 wells/group with 3 technical replicates). (G) Representative images (down) and quantification evaluation (up) of Tunel staining cells in the indicated groups (n=3 with 2 technical replicates). Red: Tunel-positive cells; blue: nuclei (Hoechst 33258). Scale bar: 100 μm. Data are presented as mean ± SEM. One-way ANOVA was used for analysis followed by Tukey's post hoc test for equal variances or Dunnett T3 post hoc test for unequal variances. $^{*}P<0.05$, $^{**}P<0.01$, $^{***}P<0.001$ compared with the BV2 group, $^{\#}P<0.05$, $^{\#\#}P<0.01$ compared with BV2+si-NC group.$^{\triangle}P<0.05$, $^{\triangle\triangle}P<0.01$, $^{\triangle\triangle\triangle}P<0.001$ compared with BV2+si-NC+LPS+ATP group.

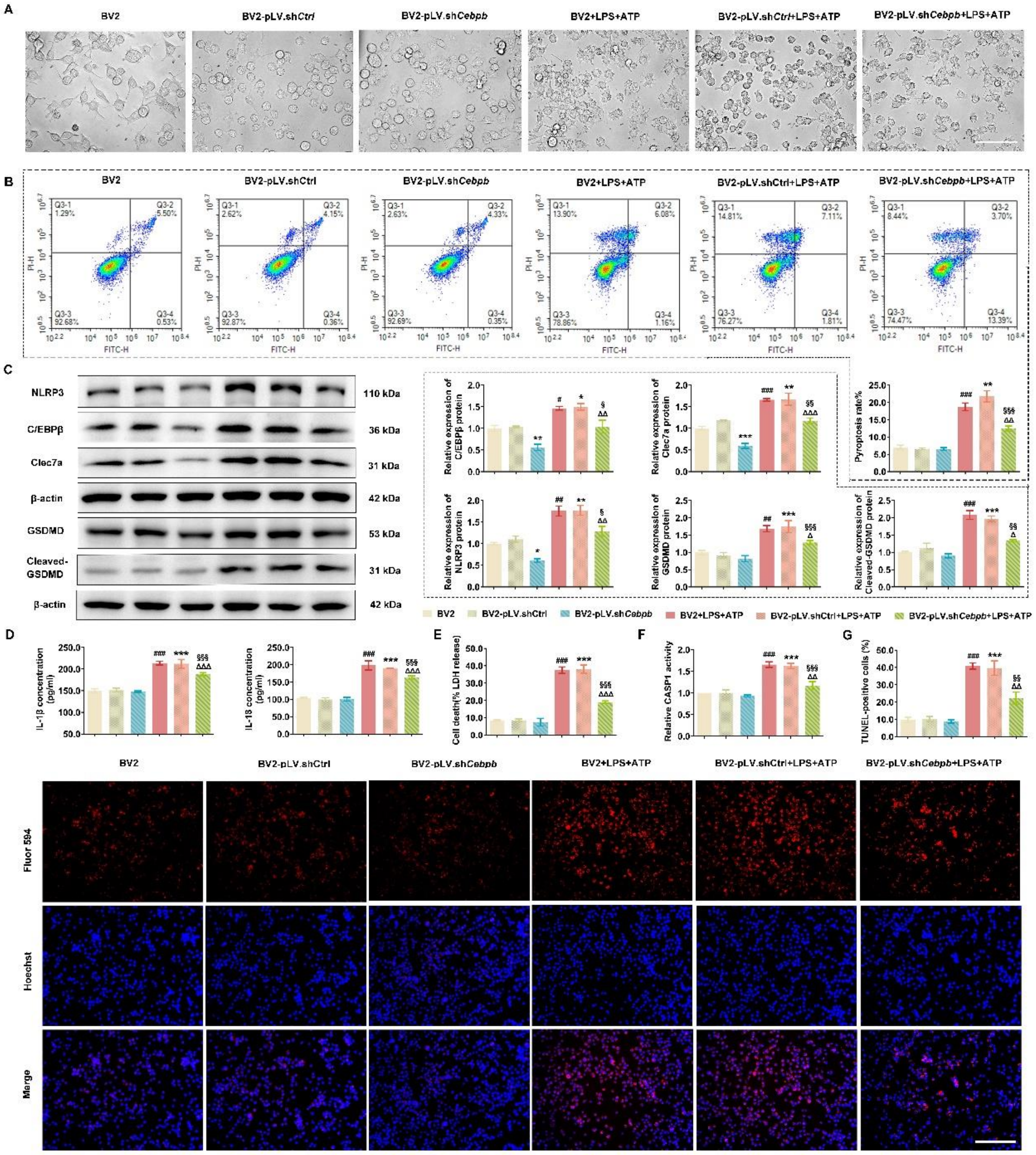

Additional file 2: Figure S2. C/EBPβ knockdown alleviated LPS and ATP induced BV2 cells pyroptosis. (A) Representative images of BV2 cells and BV2 transfected with pLV.shCtrl or pLV.sh*Cebpb* after the LPS+ATP treatments as indicated. Arrows point to the pyroptosis-like morphological changes. Scale bar: 500 μm. (B) Pyroptosis was assessed by annexin V-FITC/PI assay after incubated with LPS+ATP in indicated groups (up: representative flow cytometric dotplots; down: quantification of annexin V-FITC and PI double stained cells) (*n* = 3 wells/group with 3 technical replicates). (C)

Expression levels of pyroptosis-related proteins in cells treated with indicated components were evaluated by western blotting (*n* = 3 independent blots). (D) The concentrations of IL-1β and IL-18 in culture supernatants were assessed by ELISA (*n* = 3 wells/group with 3 technical replicates). (E) LDH release in the culture supernatants of indicated groups were determined by colorimetry (*n* = 3 wells/group with 3 technical replicates). (F) Caspase-1 activity in the cell lysis of indicated groups were determined by colorimetry (*n* = 3 wells/group with 3 technical replicates). (G) Representative images (down) and quantification evaluation (up) of Tunel staining cells in indicated groups (n=3 with 2 technical replicates). Red: Tunel-positive cells; blue: nuclei (Hoechst 33258). Scale bar: 100 μm. Data are presented as mean ± SEM. One-way ANOVA was used for analysis followed by Tukey's post hoc test for equal variances or Dunnett T3 post hoc test for unequal variances. $^{*}P<0.05$, $^{***}P<0.001$, compared with BV2 group; $^{\#}P<0.05$, $^{\#\#}P<0.01$, $^{\#\#\#}P<0.001$ compared with the BV2-pLV.shCtrl group.$^{\triangle}P<0.05$, $^{\triangle\triangle}P<0.01$, $^{\triangle\triangle\triangle}P<0.001$ compared with BV2-pLV.shCtrl+LPS+ATP group.
