# Supplementary material for: Disruption of C/EBPβ-Clec7a axis exacerbates neuroinflammatory injury via NLRP3 inflammasome-mediated pyroptosis in experimental neuropathic pain
Source: J Transl Med. 2022 Dec 12;20:583. doi: 10.1186/s12967-022-03779-9 (PMC9743596; doi:10.1186/s12967-022-03779-9)
Supplement: Supplementary file 2 — Additional file 2. Table S1. The information of siRNA and primer sequences used in this study. Table S2. The information of antibodies used in this study. Table S3. E xpression patterns of 100 di fferentially expressed genes in SC between the sham and CCI model groups by Microarray Profiling (mixed sample, n = 3). Table S4. Expression patterns of 100 differentially expressed genes in SC between the sham and CCI model groups by RNA-seq (n = 4). [file 12967_2022_3779_MOESM2_ESM.pdf]

Additional file 2: Table S1.

The information of siRNA and primer sequences used in this study

Gene	Forward (5'-3')	Reverse (5'-3')
Clec7a-Rat-qPCR	TTCGGCACTCGAAACATCCA	AGGGAGCCACCTTCTCATCT
ACTB-Rat-qPCR	ATGGATGACGATATCGCTGC	CTTCTGACCCATACCCACCA
Clec7a-siRNA	GAGUUCAUUGAAAGCCAAATT	UUUGGCUUUCAAUGAACUCTT
Cebpb-shRNA	AGAACGAGCGGCUGCAGAATT	UUCUGCAGCCGCUCGUUCUTT
si-NC	UUCUCCGAACGUGUCACGUTT	ACGUGACACGUUCGGAGAATT
Clec7a-CHIP-q1	ATGGGCCAGGGTCATCTTCC	GCAGCGAGTGAGAGCCTTC
Clec7a-CHIP-q2	TGTTCTCCTTGTTGACTTACAGC	GCACTGGCTTCATTCTTCTGGA
Clec7a-CHIP-q3	TTCGCTTTGTGTCGCTGGAG	TATAACTGCCTGGAGTCGG
GAPDH-CHIP	ACCAGGGAGGGCTGCAGTCC	TCAGTTCGGAGCCCACACGC

Additional file 2: Table S2.

The information of antibodies used in this study

Method	Antibody	Cat. No.	Dilution	Host	Reactivity	Brand	Previous publications or manufacturers
**Western blotting**	Clec7a (also known as Dectin-1)	A9883	1:1000	Rabbit	Human, Mouse, Rat	Abclonal, Wuhan, China	Tian X, Peng X, Lin J, et al. Isorhamnetin Ameliorates Aspergillus fumigatus Keratitis by Reducing Fungal Load, Inhibiting Pattern-Recognition Receptors and Inflammatory Cytokines. Invest Ophthalmol Vis Sci. 2021 Mar 1;62(3):38. PMID: 33783487; PMCID: PMC8010362. 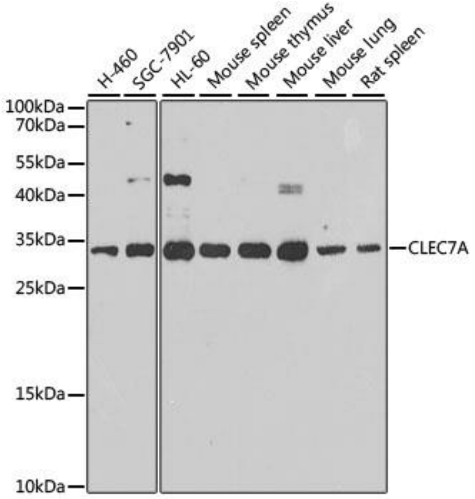

	Clec7a<br><br>(also known as Dectin-1)	ab140039	1:1000	Rabbit	Human, Rat	Abcam, MA, USA	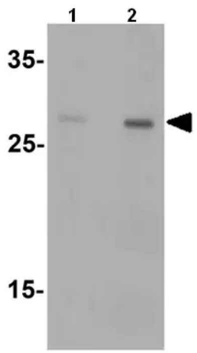<br>**Western blot - Anti-Dectin-1 antibody (ab140039)**<br><br>**Lane 1 :** Anti-Dectin-1 antibody (ab140039) at 1 µg/ml<br>**Lane 2 :** Anti-Dectin-1 antibody (ab140039) at 2 µg/ml<br><br>**All lanes :** Rat spleen tissue lysate<br><br>Lysates/proteins at 15 µg per lane.<br><br>**Predicted band size:** 28 kDa
	NLRP3	A5652	1:1000	Rabbit	Human, Mouse, Rat	Abclonal, Wuhan, China	Mi J, Yang Y, Yao H, et al. Inhibition of heat shock protein family A member 8 attenuates spinal cord ischemia-reperfusion injury via astrocyte NF-κB/NLRP3 inflammasome pathway : HSPA8 inhibition protects spinal ischemia-reperfusion injury. J Neuroinflammation. 2021 Aug 6;18(1):170. PMID: 34362408; PMCID: PMC8349068.<br>Western blot analysis of extracts of Rat lung, using NLRP3 antibody (A5652) at 1:1000 dilution.

							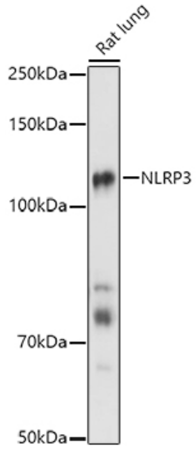
	Cleaved-GSDMD	10137S	1:1000	Rabbit	Mouse	CST, USA	Liu C, Cai B, Li D, Yao Y. Wolf-Hirschhorn syndrome candidate 1 facilitates alveolar macrophage pyroptosis in sepsis-induced acute lung injury through NEK7-mediated NLRP3 inflammasome activation. Innate Immun. 2021 Aug;27(6):437-447. PMID: 34428935; PMCID: PMC8504266.

							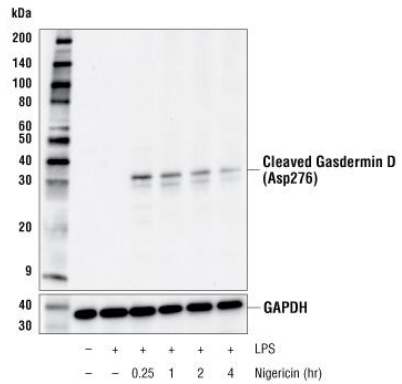
	GSDMD	ab209845	1:1000	Rabbit	Mouse, Rat	Abcam, MA, USA	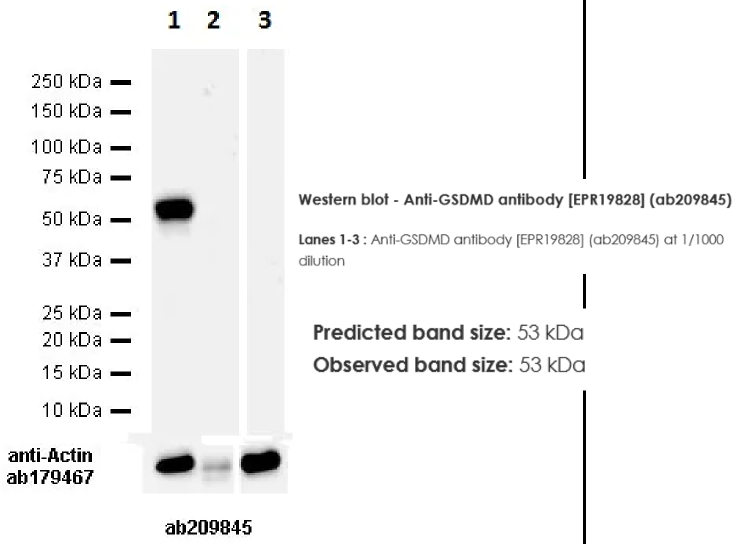

	C/EBPβ	ab32358	1:1000	Rabbit	Human, Mouse, Rat	Abcam, MA, USA	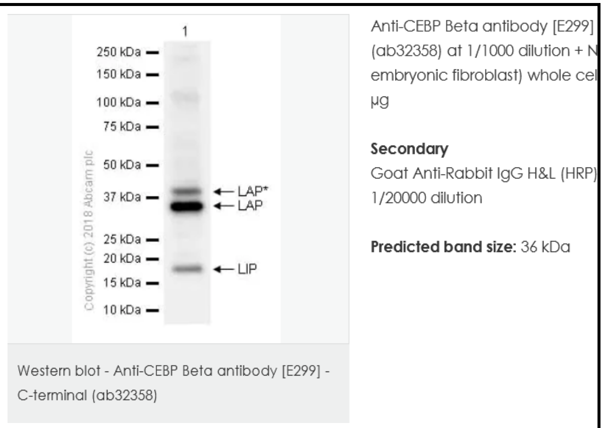 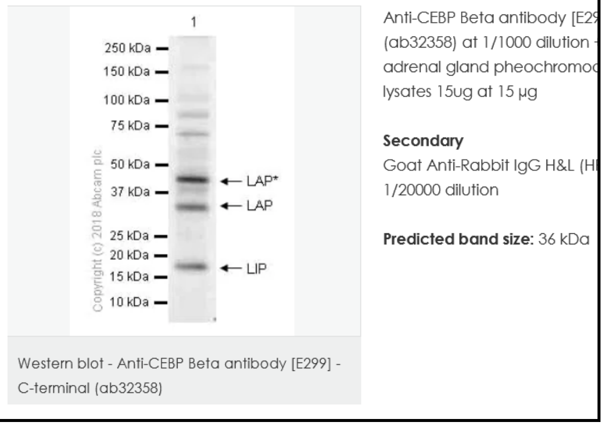

	GAPDH	ab181602	1:5000	Rabbit	Human, Mouse, Rat, Chicken, Zebrafish, African green monkey, Xenopus tropicalis	Abcam, MA, USA	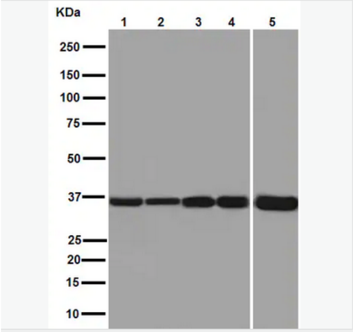<br>Western blot - Anti-GAPDH antibody [EPR16891] - Loading Control (ab181602)<br>**All lanes :** Anti-GAPDH antibo Loading Control (ab181602) a<br>**Lane 1 :** Mouse kidney lysates<br>**Lane 2 :** Mouse spleen lysates<br>**Lane 3 :** RAW 264.7 (Mouse m transformed with Abelson mu virus) whole cell lysates<br>**Lane 4 :** PC-12 (Rat adrenal g pheochromocytoma) whole<br>**Lane 5 :** Rat brain lysates<br>Lysates/proteins at 10 µg per<br>**Secondary**<br>**All lanes :** Goat Anti-Rabbit Ig (H+L),Peroxidase conjugated<br>**Predicted band size:** 36 kDa<br>**Observed band size:** 36 kDa
	β-actin	BM3873	1:2000	Rabbit	Human, Mouse, Rat, Zebrafish, Monkey	BOSTER, Wuhan, China	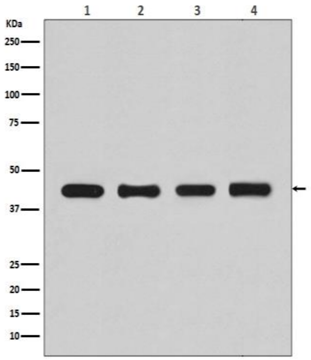<br>Western blot analysis of beta Actin expression in (1)HeLa cell lysate; (2)Human fetal kidney lysate; (3) NIH/3T3 cell lysate; (4) PC-12 cell lysate.

	pSyk	MA5-14918	1:1000	Rabbit	Human, Mouse, Rat	Thermo Scientific, CA, USA	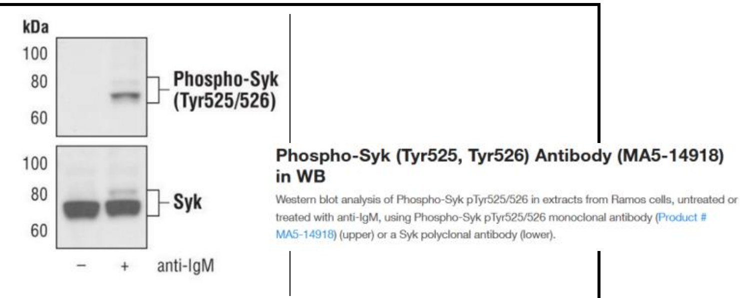
	Syk	D3Z1E	1:1000	Rabbit	Human, Mouse, Rat	CST, USA	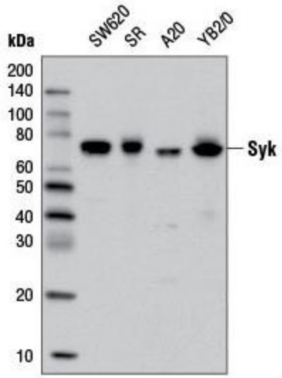
	pERK	P63086	1:1000	Rabbit	Human, Mouse, Rat	RayBiotech USA	Volmer, Romain et al. "Borna disease virus blocks potentiation of presynaptic activity through inhibition of protein kinase C signaling." PLoS pathogens vol. 2,3 (2006): e19. PMID: 16552443; PMCID: PMC1401496.

							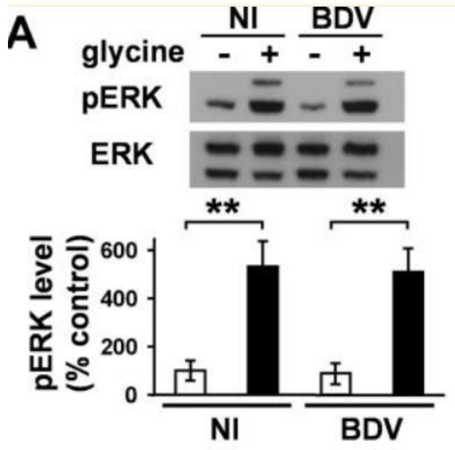
	ERK	A4782	1:1000	Rabbit	Human, Mouse, Rat	Abclonal, Wuhan, China	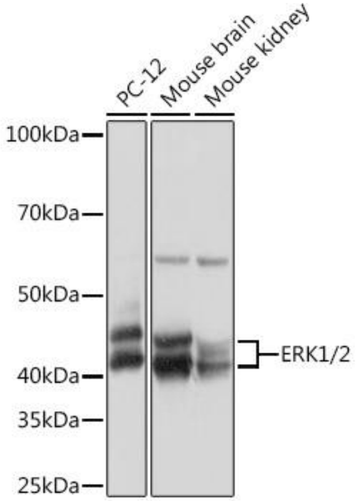
	pJNK	AP0276	1:1000	Rabbit	Human, Mouse, Rat	Abclonal, Wuhan, China	Western blot analysis of extracts from C6 cells untreated or treated with anisomycin using Phospho-JNK1-T183/Y185 + JNK2-T183/Y185 + JNK3-T221/Y223 Antibody (AP0276).

							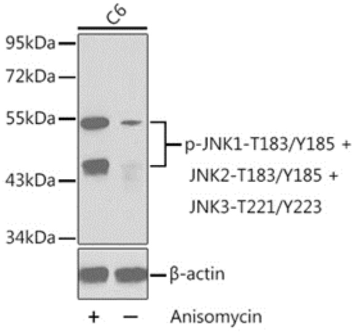
	JNK	ab179461	1:1000	Rabbit	Human, Mouse, Rat, Chicken, Cow, Dog, Zebrafish, African green monkey, Xenopus tropicalis	Abcam, MA, USA	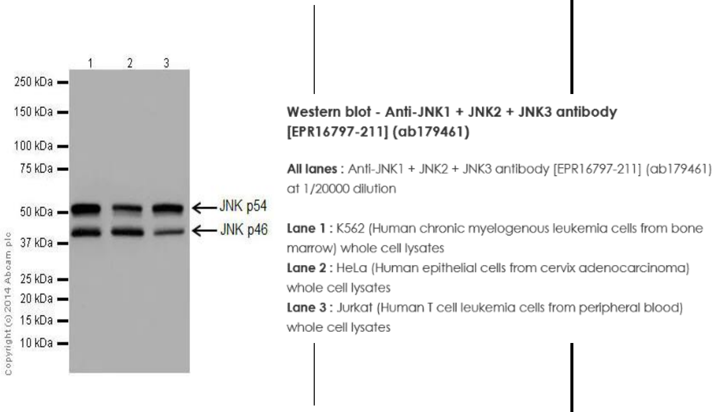

**Immunochemistry**	Clec7a	ab140039	1:100	Rabbit	Human, Rat	Abcam, MA, USA	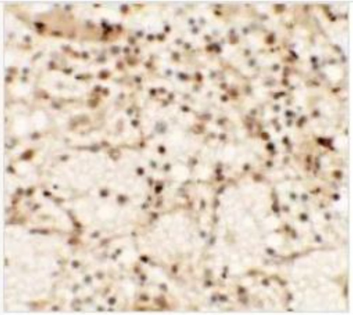 Immunohistochemistry (Formalin/PFA-fixed paraffin-embedded sections) - Anti-Dectin-1 antibody (ab140039)
	C/EBPβ	MA1-827	1:200	Mouse	Mouse, Rat	Thermo Scientific, CA, USA	Liu Q, Zhu Y, Yong WK, et al. Cutting Edge: Synchronization of IRF1, JunB, and C/EBPβ Activities during TLR3-TLR7 Cross-Talk Orchestrates Timely Cytokine Synergy in the Proinflammatory Response. J Immunol. 2015 Aug 1;195(3):801-5. PMID: 26109639; PMCID: PMC4505950.

**Immunofluorescence**	Clec7a	ab140039	1:50	Rabbit	Human, Rat	Abcam, MA, USA	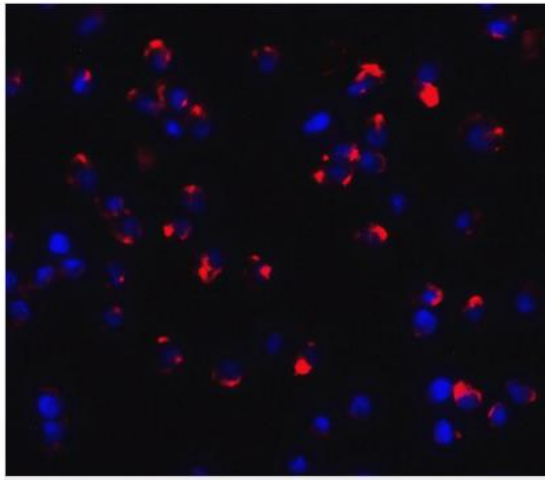 Immunocytochemistry/ Immunofluorescence - Anti-Dectin-1 antibody (ab140039)
	NLRP3	AF2155	1:50	Rabbit	Human, Mouse, Rat	Beyotime, Shanghai, China	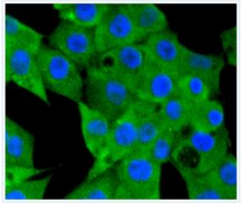 Fig. 1. ICC staining NLRP3 in PMVEC cells (green). The nuclear counter stain is DAPI (blue).Cells were fixed in paraformaldehyde, permeabilised with 0.25% Triton X-100/PBS. 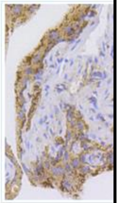 Fig. 2. Immunohistochem embedded mouse bladde antibody. Counter stained
	Iba-1	ab283319	1:100	Mouse	Human, Mouse, Rat	Abcam, MA, USA	

							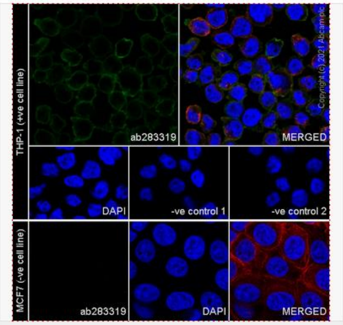<br><br>Immunocytochemistry/ Immunofluorescence - Anti-Iba1 antibody [EPR16589] - Chimeric (ab283319)<br>Immunofluorescent analysis of<br>Paraformaldehyde-fixed, 0.1%<br>permeabilized THP-1 cells label<br>ab283319 at 1/100 (11.36 ug/m<br>followed by ab150113 Goat An<br>H&L (Alexa Fluor® 488) antibod<br>dilution (Green). ab179513 Anti<br>rabbit monoclonal antibody w<br>counterstain tubulin at 1/200 di<br>Nuclear counterstain was DAPI<br>image showing cytoplasmic st<br>cells.<br>**Negative control**: MCF7<br>Secondary antibody only cont<br>antibody is ab150113 Goat Ant<br>(Alexa Fluor® 488) at 1/1000 dil<br>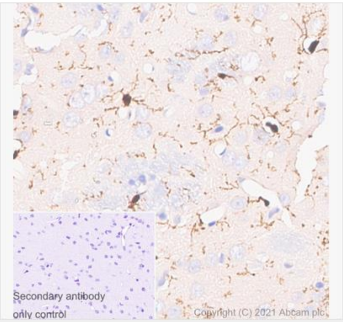<br><br>Immunohistochemistry (Formalin/PFA-fixed paraffin-embedded sections) - Anti-Iba1 antibody [EPR16589] - Chimeric (ab283319)<br>Immunohistochemical analysis<br>embedded Rat cerebrum tissu<br>with ab283319 at 1/10000 (0.11<br>followed by a ready to use Lei<br>(Bond™ Polymer Refine Detect<br>staining on the microglia in rat<br>section was incubated with ab<br>mins at room temperature and<br>mouse specific IgG antibody (<br>mins. The immunostaining was<br>Leica Biosystems BOND® RX ins<br>Counterstained with Hematoxy<br>Secondary antibody only cont<br>antibody is a ready to use Leic<br>Polymer Refine Detection).<br>Heat mediated antigen retriev<br>buffer (pH 6.0, epitope retrieva<br>mins
	GFAP	3670	1:100	Mouse	Human, Mouse, Rat	CST, USA	Leinster VHL, Phillips TJ, et al. Cortical cells are altered by factors including bone morphogenetic protein released from a placental barrier model under altered oxygenation. Neuronal Signal. 2020 Apr 9;4(1):NS20190148. PMID: 32714599; PMCID: PMC7363303.

							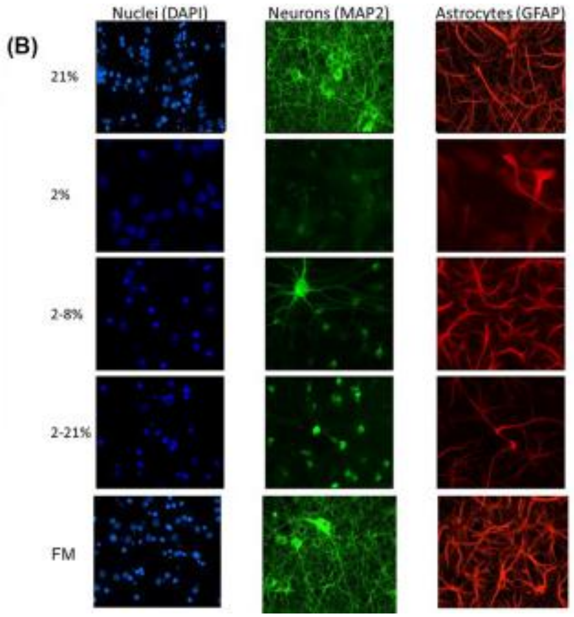
	NeuN	ab104224	1:100	Mouse	Human, Mouse, Rat	Abcam, MA, USA	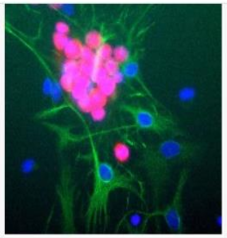<br>Immunocytochemistry - Anti-NeuN antibody [1B7] - Neuronal Marker (ab104224)<br>Rat brain neural cultures sta in pink, with ab4674 (chicke GFAP) in green and DNA in reveals strong nuclear and staining. It does not stain ast non-neuronal cells.

<table>
<tr>
<td rowspan="2"></td>
<td>Goat Anti-Mouse IgG H&L (Alexa Fluor® 488)</td>
<td>ab150113</td>
<td>1:500</td>
<td>Goat</td>
<td>Mouse</td>
<td>Abcam, MA, USA</td>
<td>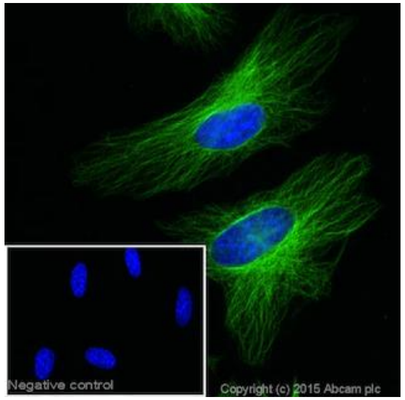
</td>
</tr>
<tr>
<td>Goat Anti-Rabbit IgG H&L (Cy3 ®) preadsorbed</td>
<td>ab6939</td>
<td>1:500</td>
<td>Goat</td>
<td>Rabbit</td>
<td>Abcam, MA, USA</td>
<td>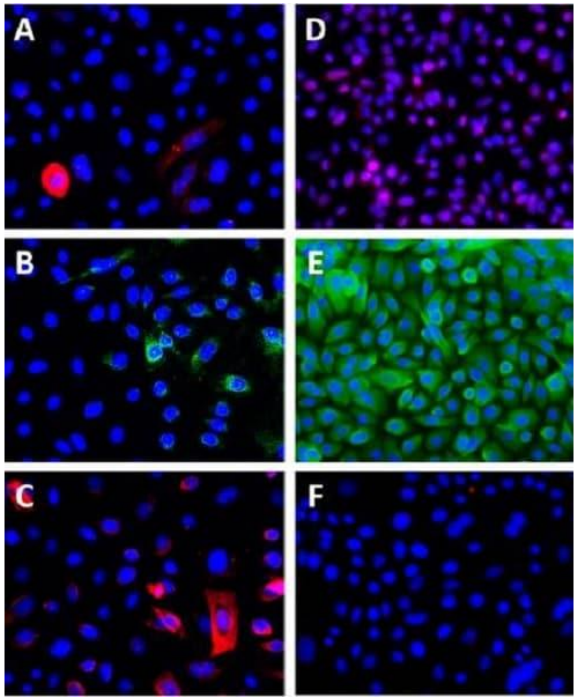
</td>
</tr>
</table>

Additional file 2: Table S3. Expression patterns of 100 differentially expressed genes in SC between the sham and CCI model groups by Microarray Profiling (mixed sample, n=3)

Gene Symbol	SC-Fold change (Mod/ Sham)	|SC-log2 (Sham/Mod)|	Dysregulation Patterns
Cxcl13	12.63686944	3.6595672	Up
Reg3b	10.453195	3.385872	Up
Clec7a	6.5039538	2.701317	Up
Ccl2	4.3116737	2.108248	Up
Vom2r7	0.291312821	1.779358896	Down
Hamp	3.2359807	1.695203	Up
Olr1169	0.308880777	1.694878007	Down
Olr1564	0.317040617	1.657260416	Down
Usp18	0.319336401	1.646851079	Down
Olr1052	0.33372207	1.583280996	Down
Olr1274	0.33454991	1.57970664	Down
Sprr1a	2.8110654	1.491117	Up
Expi	0.36093	1.470209032	Down
Irf7	0.36204	1.465779	Down
Apold1	0.3656763	1.451361	Down

Isg15	0.3904915	1.356637	Down
Il18rap	2.490318	1.31633	Up
Serpina3n	2.458571	1.29782	Up
Gal	2.4365891	1.284863	Up
Alb	0.4133971	1.2744	Down
Oas1i	0.4289769	1.221028	Down
Usp18	0.4331921	1.206921	Down
Csrp3	2.2698044	1.182568	Up
Atf3	2.1963774	1.135126	Up
Tslp	2.1824128	1.125924	Up
LOC681283	2.1732829	1.119876	Up
RT1-Da	2.1197704	1.083908	Up
Mx2	0.4950064	1.014481	Down
Rtp4	0.5025026	0.992797	Down
RT1-Ba	1.987886	0.991235	Up
Reg3a	1.984784	0.988982	Up
Cd74	1.9775223	0.983694	Up
Serpina1	1.9683953	0.97702	Up

A2ld1	1.9285987	0.947553	Up
Stc1	0.5318974	0.91078	Down
RGD1309808	0.5326796	0.90866	Down
Cx3cr1	1.8650599	0.899222	Up
Fxyd5	1.8557289	0.891986	Up
Ntng1	0.5412537	0.885623	Down
Gpr34	1.8366847	0.877104	Up
LOC100360522	1.8355215	0.87619	Up
LOC100911291	1.8192603	0.863352	Up
Smad9	0.5523773	0.856274	Down
Nfam1	1.8035795	0.850863	Up
Apobec1	1.79721	0.845759	Up
LOC681204	1.7841081	0.835203	Up
LOC100363064	1.7760596	0.82868	Up
Slc16a14	0.5635342	0.827425	Down
Ifit1	0.5682186	0.815482	Down
RT1-DMb	1.7570217	0.813132	Up
Tyrobp	1.7412616	0.800133	Up

Oasl	0.578894	0.788629	Down
RGD1566383	1.7085885	0.772805	Up
Oasl2	0.5944713	0.750321	Down
RGD1560815	1.6582528	0.729664	Up
Ifit2	0.6073104	0.719494	Down
Lyz2	1.6418758	0.715345	Up
Rac2	1.6367909	0.71087	Up
Tubb6	1.636767	0.710849	Up
LOC686123	1.6361023	0.710263	Up
Csf2rb	1.6313236	0.706043	Up
LOC679711	0.6148639	0.701661	Down
Itgam	1.6250364	0.700472	Up
Il18	1.6232228	0.698861	Up
Sidt1	0.6184869	0.693185	Down
Ifih1	0.6239849	0.680417	Down
Pou2f2	1.5997816	0.677875	Up
Mrgprb4	0.6255842	0.676724	Down
Pglyrp3	0.626179	0.675353	Down

Dapp1	1.5878845	0.667106	Up
Kcnk18	0.6309869	0.664318	Down
Csf1r	1.5837153	0.663313	Up
Rpe65	0.6347714	0.655691	Down
Fcgr2a	1.5695304	0.650333	Up
Tfec	1.5676582	0.648611	Up
P2ry13	1.5618761	0.64328	Up
Ly49s3	0.6441654	0.634497	Down
Ms4a11	1.5475049	0.629944	Up
Lcp1	1.5442957	0.626949	Up
Timp1	1.5412854	0.624134	Up
LOC310177	1.5360967	0.619269	Up
Vav1	1.5355654	0.61877	Up
Olr1069	1.5335562	0.616881	Up
Tgfbr1	1.5288512	0.612448	Up
Cxcl10	0.6558328	0.6086	Down
Cd4	1.5230085	0.606924	Up
LOC685987	1.5179697	0.602143	Up

Magee2	0.6592484	0.601106	Down
Ddx58	0.6592977	0.600998	Down
Ccr5	1.5164038	0.600654	Up
Fam111a	0.6603441	0.59871	Down
Anxa3	1.5125858	0.597017	Up
Pcsk2	0.66186	0.595402	Down
Clec2d	1.5085693	0.593181	Up
Ptpn6	1.5070246	0.591703	Up
RGD1561086	1.5049452	0.589711	Up
Gbp4	0.6649317	0.588722	Down
Olr551	1.5035689	0.588391	Up
RGD1564324	1.5021126	0.586993	Up
Laptm5	1.5007243	0.585659	Up

Additional file 2: Table S4. Expression patterns of 100 differentially expressed genes in SC between the sham and CCI model groups by RNA-seq (n=4)

Gene Symbol	Sham-average	CCI-average	Fold change(Model/Sham)	Regulation	P.Value
Nlrp3	7.25	360	49.6552	Up	0.027217
Ddx17	158.75	6281	39.5654	Up	0.020881954
Cacna2d1	6	181.25	30.2083	Up	0.026828546
Limk1	4417	253	-17.4585	Down	0.000366165
Dock6	27	389.5	14.4259	Up	0.026711804
Atf3	61.25	794.25	12.9673	Up	0.002799736
Clec7a	21	257	12.2381	Up	0.008697782
Slc41a3	89.75	7.25	-12.3793	Down	0.00122233
Ccl2	19.25	210.5	10.9351	Up	0.014396435
Wdr18	111.75	11	-10.1591	Down	0.022777859
Gpr3	13	111	8.5385	Up	0.025625662
Cxcl13	69.5	549	7.8993	Up	0.007413509
Reg3b	112.5	726.5	6.4578	Up	0.00327874
Atf1	234.25	36.25	-6.4621	Down	0.04522935
Trmt5	561	90.75	-6.1818	Down	0.036085811

Aoah	19.25	113.75	5.9091	Up	0.010274523
RT1 DMb	4.25	24.5	5.7647	Up	0.000365273
Abcg3l1	8.25	46.75	5.6667	Up	0.001681187
Pold2	14	75.5	5.3929	Up	0.034123136
Phldb2	86	432.5	5.0291	Up	0.01349678
Adgrg5	13	64.5	4.9615	Up	0.003349098
Ank2	25.25	124.75	4.9406	Up	0.006499653
Aifm1	264.75	1303	4.9216	Up	0.000117058
LOC100910270	253.25	1243.5	4.9102	Up	0.000578777
AABR07025896.1	2.5	12.25	4.9	Up	0.010337416
Il18rap	40.25	192.5	4.7826	Up	0.000546411
Mis18bp1	6	28.25	4.7083	Up	0.033601633
Dock11	300.75	1389.5	4.6201	Up	0.024427212
Aldh3a1	3.5	16	4.5714	Up	0.005282585
Tfec	17.5	78.25	4.4714	Up	0.014863585
Stpg1	17.5	75.5	4.3143	Up	0.013288392
Tmem144	57.75	242.75	4.2035	Up	0.018069545
Trpc6	137.25	576	4.1967	Up	0.001008163

Acpp	16.75	69.5	4.1493	Up	0.001260544
Clec12a	101.25	370	3.6543	Up	0.000377991
Scimp	4.5	16.25	3.6111	Up	0.002667134
Il6	13.25	47.75	3.6038	Up	0.00044409
3-Mar	5.5	19.75	3.5909	Up	0.025505106
Lrrc20	47.25	169.5	3.5873	Up	0.034298421
Csf2rb	357	1280.5	3.5868	Up	0.001546693
Tspan9	847.75	3015	3.5565	Up	0.012887657
Traf3	2.25	8	3.5556	Up	0.002207126
Mmp10	8	28.25	3.5312	Up	0.04965938
Fcnb	16	55.25	3.4531	Up	0.001444493
Mtmr1	543	1871.25	3.4461	Up	0.02559201
Grhl3	45.75	156.5	3.4208	Up	0.006936756
Cd6	24.25	82.75	3.4124	Up	0.029934899
Hamp	13	44.25	3.4038	Up	0.004685636
C4b	907	3067	3.3815	Up	0.000380506
LOC103689965	3579.5	11982.75	3.3476	Up	3.47917E-05
Pax8	2.25	7.25	3.2222	Up	0.037499916

Cfd	2.25	7.25	3.2222	Up	0.040493553
Hcst	9.25	29.75	3.2162	Up	0.013186353
Tmem217	2.5	8	3.2	Up	0.047459447
RGD1561662	34	108.5	3.1912	Up	0.039741663
Dlg3	2752.25	866.5	-3.1763	Down	0.014387746
Rps24	2621	8307	3.1694	Up	0.044079443
Nrg1	15	47	3.1333	Up	0.001210881
Ms4a7	5	15.5	3.1	Up	0.046421082
Cdkl3	136.5	417.75	3.0604	Up	0.016127996
Filip1	1.75	5.25	3	Up	0.041390044
Epb41	0.5	1.5	3	Up	0.049074512
Matn1	23.75	8	-2.9688	Down	0.000200369
Anxa3	256.5	754.5	2.9415	Up	0.001936808
Jup	47	138	2.9362	Up	0.032936928
AABR07043115.1	6.75	19.75	2.9259	Up	0.025979578
AC132720.2	6.5	19	2.9231	Up	0.042788415
Il1b	25.75	74.75	2.9029	Up	0.015322608
C1qc	2126.75	6168.25	2.9003	Up	0.000312191

Dmgdh	7	20.25	2.8929	Up	0.006303412
Bpifb5	2.25	6.5	2.8889	Up	0.047855506
Atp2b1	9509.75	3309.5	-2.8735	Down	0.014597784
Itgal	587.25	1685	2.8693	Up	0.000126381
Lilrb3a	7	20	2.8571	Up	0.01221508
Glmp	10.5	29.5	2.8095	Up	0.047111067
LOC108348139	5	14	2.8	Up	0.007024643
Brca1	151.25	54.25	-2.788	Down	0.006089391
Aifm1	1799.75	652.75	-2.7572	Down	0.000784195
F10	39.25	108	2.7516	Up	0.001141333
LOC689064	40.25	110.75	2.7516	Up	0.023909017
Slc5a10	2	5.5	2.75	Up	0.039849286
Cyp26c1	7.75	21.25	2.7419	Up	0.009751576
C3	12875.25	34705	2.6955	Up	0.000363328
Lepr	94	250.25	2.6622	Up	0.0487574
Unc13d	75.25	200.25	2.6611	Up	0.01377725
Mefv	113.25	300	2.649	Up	0.001320787
Nol8	6.25	16.5	2.64	Up	0.03543862

Sctr	10.75	28.25	2.6279	Up	0.009390802
Ms4a6bl	44.25	115.5	2.6102	Up	0.013480328
Sag	10.25	26.75	2.6098	Up	0.038420386
Ddah2	42.25	109.5	2.5917	Up	0.018251581
Tlr8	66	169.5	2.5682	Up	0.012424392
RT1 A2	531.5	1358.25	2.5555	Up	0.010026734
Xpnpep1	1988	781	-2.5455	Down	0.033369082
C1qa	4373.25	10969.25	2.5083	Up	6.79946E-05
Cxcl6	7.5	18.75	2.5	Up	0.0319984
AABR07039806.1	10	25	2.5	Up	0.045150879
Mslnl	22	55	2.5	Up	0.000416436
Opn1sw	5.75	14.25	2.4783	Up	0.016900877
Ppp1r15a	177.25	439	2.4767	Up	0.00129702
